# Plasma Concentrations, Efficacy and Safety of Efavirenz in HIV-Infected Adults Treated for Tuberculosis in Cambodia (ANRS 1295-CIPRA KH001 CAMELIA Trial)

**DOI:** 10.1371/journal.pone.0090350

**Published:** 2014-03-07

**Authors:** Laurence Borand, Yoann Madec, Didier Laureillard, Monidarin Chou, Olivier Marcy, Phearavin Pheng, Narom Prak, Chindamony Kim, Khemarin Kim Lak, Chanroeun Hak, Bunnet Dim, Eric Nerrienet, Arnaud Fontanet, Thim Sok, Anne E. Goldfeld, François-Xavier Blanc, Anne-Marie Taburet

**Affiliations:** 1 Institut Pasteur du Cambodge, Epidemiology and Public Health Unit, Phnom Penh, Cambodia; 2 Institut Pasteur, Unité de Recherche et d'Expertise Epidémiologie des Maladies Emergentes, Paris, France; 3 ANRS, Ho Chi Minh City, Vietnam; 4 Faculty of Pharmacy, University of Health Sciences, Phnom Penh, Cambodia; 5 Khmer-Soviet Friendship Hospital, Phnom Penh, Cambodia; 6 Donkeo Provincial Hospital, Takeo, Cambodia; 7 Médecins Sans Frontières, Phnom Penh, Cambodia; 8 Svay Rieng Provincial Hospital, Svay Rieng, Cambodia; 9 Cambodian Health Committee, Phnom Penh, Cambodia; 10 Calmette Hospital, Phnom Penh, Cambodia; 11 Siem Reap Referral Hospital, Siem Reap, Cambodia; 12 Institut Pasteur du Cambodge, HIV/Hepatitis Laboratory, Phnom Penh, Cambodia; 13 Conservatoire National des Arts et Métiers, Paris, France; 14 Program in Cellular and Molecular Medicine, Children's Hospital, Harvard Medical School, Boston, Massachusetts, United States of America; 15 Assistance Publique - Hôpitaux de Paris, Hôpital Bicêtre, Hôpitaux Universitaires Paris Sud, Pneumology Department, Le Kremlin-Bicêtre, France; 16 UMR INSERM 1087 CNRS UMR_6291, l′Institut du Thorax, Service de Pneumologie, CHU de Nantes, DHU2020, Université de Nantes, France; 17 Assistance Publique - Hôpitaux de Paris, Hôpital Bicêtre, Hôpitaux Universitaires Paris Sud, Clinical Pharmacy Department, Le Kremlin-Bicêtre, France; Asociacion Civil Impacta Salud y Educacion, Peru

## Abstract

**Objective:**

To assess efavirenz plasma concentrations and their association with treatment efficacy and tolerance of efavirenz 600 mg daily in HIV-tuberculosis co-infected patients.

**Methods:**

HIV-infected adults with CD4+ T cell count ≤200/mm^3^ received standard 6-month tuberculosis treatment and antiretroviral therapy including a daily-dose of 600 mg of efavirenz, irrespective of their body weight. Mid-dose blood samples were drawn both on tuberculosis treatment (week +2 and week +6 after antiretroviral therapy initiation, and week 22 of follow-up) and off tuberculosis treatment (week 50 of follow-up). Considered therapeutic range was 1,000 to 4,000 ng/mL. Multivariate analysis was performed to evaluate the association between efavirenz concentration below 1,000 ng/mL and virological failure. Linear regression was used to test the association between efavirenz exposure and CD4+ T cell gain. Severe side effects potentially related to efavirenz were described and their association with efavirenz exposure was tested by multivariate analysis.

**Results:**

Efavirenz plasma concentrations were available in 540 patients. Median [interquartile range] efavirenz concentrations were 2,674 ng/mL [1,690–4,533], 2,667 ng/mL [1,753–4,494] and 2,799 ng/mL [1,804–4,744] at week +2, week +6, week 22, respectively, and 2,766 ng/mL [1,941–3,976] at week 50. Efavirenz concentrations were lower at week 50 (off rifampicin) compared to week 22 (on rifampicin) (p<0.001). Late attendance to study visit and low hemoglobinemia were the only factors associated with an increased risk of efavirenz concentration below 1,000 ng/mL. Efavirenz concentration below 1,000 ng/mL was not associated with treatment failure. Efavirenz concentration above 4,000 ng/mL was associated with higher risk of central nervous system side effects (p<0.001) and of hepatotoxicity (p<0.001).

**Conclusion:**

Body weight and tuberculosis treatment were not associated with low efavirenz concentrations or treatment failure, supporting the 600 mg daily-dose of efavirenz in HIV-tuberculosis co-infected patients. High efavirenz concentrations were related to a higher risk of central nervous system side effects and hepatotoxicity.

**Trial Registration:**

ClinicalTrials.gov NCT01300481

## Introduction

Efavirenz is a non-nucleoside reverse transcriptase inhibitor (NNRTI) widely used in combination with nucleoside reverse transcriptase inhibitors as first-line treatment of HIV-1 infection. Concomitant administration of antiretroviral therapy (ART) and rifampicin - a potent inducer of drug metabolizing enzymes - is challenging due to drug-drug interactions [1]. Efavirenz-containing ART is the first-line treatment recommended by the World Health Organization (WHO) in HIV-infected patients, especially when treated concurrently with tuberculosis treatment including rifampicin and isoniazid for 6 months and ethambutol and pyrazinamide for the first 2-months [2]. However, the appropriate dose of efavirenz during rifampicin-based tuberculosis treatment remains debated [3], [4]. Some guidelines recommend increasing efavirenz dosing to 800 mg daily when patient body weight is above 50 kg [5], [6] or above 60 kg [7], [8] while the WHO and U.S. Centers for Disease Control and Prevention recommend maintaining a 600 mg daily dose, irrespective of patient's body weight [9], [10].

The CAMELIA (ANRS 1295-CIPRA KH001) randomized clinical trial showed a 34% reduction of mortality in severely immunocompromised HIV-infected adults treated for tuberculosis when efavirenz-containing ART was initiated two weeks compared to eight weeks after tuberculosis treatment onset [11]. Here, we describe plasma concentrations of efavirenz over one year of follow-up on and off tuberculosis treatment in 540 patients included in the CAMELIA trial. We also investigated risk factors associated with efavirenz concentrations below the therapeutic range and we analyzed the association between efavirenz exposure and efficacy and toxicity.

## Materials and Methods

The protocol of the CAMELIA trial and the TREND statement check list of this longitudinal pharmacological study are available as supporting information; see CAMELIA Protocol S1 and TREND Statement Checklist S1.

### Ethical considerations

The CAMELIA trial was approved by the Cambodian National Ethics Committee and by the Institutional Review Boards of the Immune Disease Institute of Harvard Medical School and Médecins Sans Frontières. This trial was conducted in accordance with the Declaration of Helsinki [12] and all patients signed the informed consent form prior to inclusion. The CAMELIA trial is registered with ClinicalTrials.gov, number NCT00226434.

### Study population and treatments

Main characteristics of enrolled patients and trial design have been described elsewhere [11]. Among the 661 ART-naïve, HIV-infected adults with CD4+ T cell count ≤200/mm^3^ and newly diagnosed, smear-positive tuberculosis who were recruited in CAMELIA and followed in five Cambodian hospitals from 2006 to 2010, we conducted a longitudinal pharmacological study in some of them. Tuberculosis treatment consisted of a six-months regimen containing rifampicin (8–10 mg/kg/day), isoniazid (4–5 mg/kg/day), ethambutol (15–20 mg/kg/day) and pyrazinamide (20–30 mg/kg/day) as fixed dose combination (FDC) for the first two months, followed by rifampicin (8–10 mg/kg/day) and isoniazid (4–5 mg/kg/day) as FDC for the next four months. In the CAMELIA trial, patients were randomized to initiate ART either two weeks (“early-ART” group) or eight weeks (“late-ART” group) after onset of tuberculosis treatment. ART consisted of lamivudine (150 mg) and stavudine (30 mg) twice daily and efavirenz 600 mg once daily in the evening, irrespective of patients' body weight.

### Blood samples and efavirenz assay

**Figure 1:**
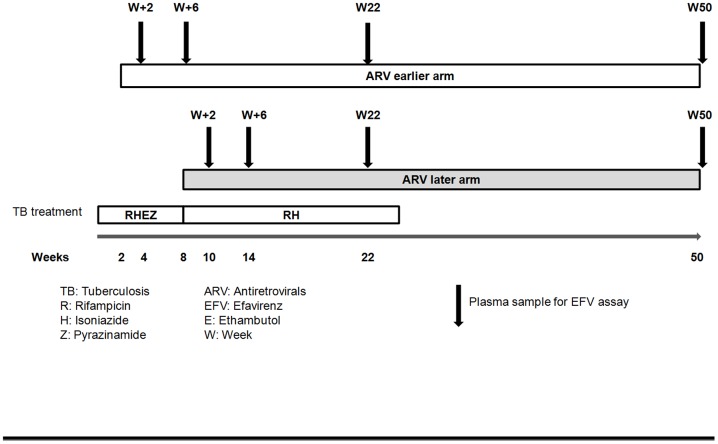
Study design.

To account for the differential timing of ART initiation in the two study arms, blood samples for determination of plasma efavirenz concentration were drawn two weeks (W+2) and six weeks (W+6) after ART initiation for all patients, corresponding to four and eight weeks after tuberculosis treatment onset in the early-ART group, and 10 and 14 weeks after tuberculosis treatment onset in the late-ART group. Follow-up blood samples were also drawn at week 22 and week 50 after tuberculosis treatment onset in both groups. At this latter time point, patients received efavirenz without concomitant tuberculosis treatment (**Figure 1**).

Blood samples were collected early in the morning on EDTA tubes 14±2 hours after efavirenz intake and reflected mid-dosing plasma concentrations [13], [14]. After centrifugation, plasma was transferred and stored at −80°C until analysis. Efavirenz was assayed in plasma samples at the University of Health Sciences in Phnom Penh, Cambodia, using a validated high-performance liquid chromatography method with UV detection at 248 nm [15]. The lower limit of quantification was 50 ng/mL. All samples with concentration below this limit were removed from analysis as these patients were considered as not taking efavirenz. The laboratory participated to blind external quality controls provided by Asqualab (France). Within and between days variability of the assay - estimated from quality control samples inserted in each analytical run - were below 15%. The therapeutic range of efavirenz was considered to be 1,000–4,000 ng/mL, as previously reported [14].

### Efficacy markers and adverse events recorded

Virological efficacy of ART was assessed at week 8 in patients from the early arm and at week 26 and week 50 for all patients. It was described as the proportion of patients with undetectable plasma HIV RNA (<250 copies/mL). CD4+ T cell gain since ART initiation at week 26 and week 50 was considered for immunological reconstitution in all patients. Clinical or biological adverse events were reported and graded by on-site investigators using the Division of AIDS table for grading the severity of adults and pediatric adverse events [16]. All events were coded using the MedDRA thesausus. Events of interest for this study were selected in the following categories from the package insert of Sustiva [17]: central nervous system (CNS) manifestations, elevated liver enzymes, cutaneous manifestations. They were analyzed until week 58. The number of side effects was reported by type and severity grade.

### Statistical analysis

Efavirenz plasma concentrations on and off rifampicin-based tuberculosis treatment were described as median and inter quartile range [IQR] and were compared at two consecutive time-points using paired Wilcoxon tests. Factors associated with efavirenz concentration below 1,000 ng/mL were identified using a mixed effect logistic regression to account for multiple measurements within patients. The main exposure variables were concomitant tuberculosis treatment and patients' body weight at the time of sampling.

We evaluated the association between plasma concentrations of efavirenz and: i) virological efficacy, defined as a viral load <250 copies/mL at week 8 (only considering patients from the early arm), week 26 and week 50 of follow-up, using a logistic regression; ii) immunological efficacy, defined as the CD4+ T cell gain at week 26 and week 50 of follow-up, using linear regression models; and iii) occurrence of adverse events before week 58 of follow-up using a logistic regression. Only plasma concentrations of efavirenz prior to the evaluation time were considered. The threshold in efavirenz concentrations was 1,000 ng/mL for the association with virological and immunological efficacy, and 4,000 ng/mL for the association with occurrence of adverse events. Other factors considered in all these analyses were the study site, CAMELIA study arm, gender, age at enrolment, body mass index (BMI) and body weight as recorded at ART initiation and sampling time, tuberculosis location, occurrence of paradoxical tuberculosis-associated immune reconstitution inflammatory syndrome (IRIS), as well as biological factors measured at ART initiation (defined as the closest measurement within the month preceding ART initiation for CD4+ T cells count, HIV RNA concentration, hemoglobin, transaminases) and/or at the sampling time (week 2±2 weeks, week 8±4 weeks, week 22±4 weeks and week 50±8 weeks).

In all analyses, factors associated with a p-value <0.20 in univariate analysis were entered in the multivariate model. Factors independently associated with the outcome were then identified using a stepwise backward procedure based on the Wald test. Two-sided hypotheses and tests were adopted for all statistical inferences, a p-value <0.05 being considered statistically significant. All statistical analyses were performed using STATA 11 software (Stata Corp., College Station, Texas, USA).

## Results

### Study population

**Figure 2:**
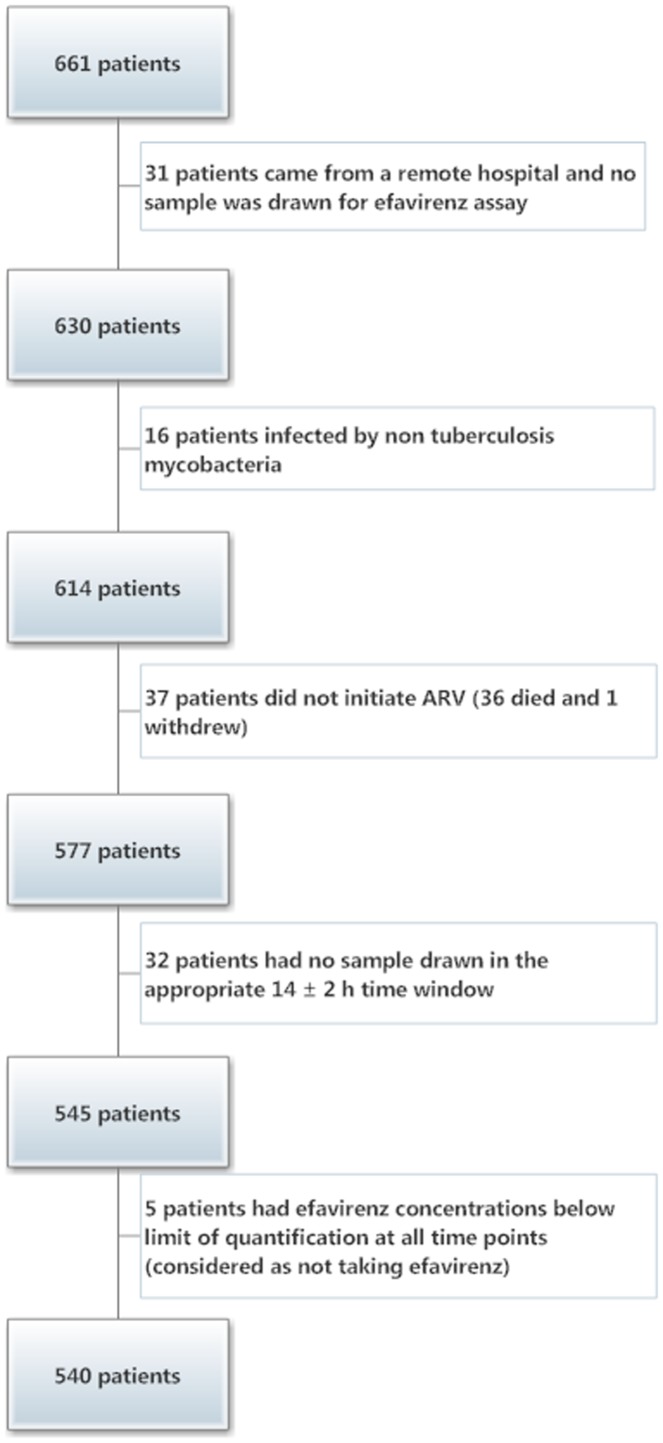
Study population.

Among the 661 patients enrolled in the CAMELIA trial, 540 were considered in this analysis. The remaining 121 patients were not included for various reasons shown in **Figure 2** **.** Characteristics of the patients at ART initiation are displayed in **Table 1** **.** Patients were at an advanced AIDS stage with a median [IQR] CD4+ T cell count of 25 cells/mm^3^ [12–60] and median [IQR] BMI of 17.3 kg/m^2^ [15.8–19.1]; 167 patients (31%) had a body weight ≥50 kg, including 27 (5%) patients weighing ≥60 kg. The number of patients with body weight ≥50 kg increased during the study, reaching 261 (58.1%) and 277 (68.7%) at week 22 and week 50, respectively. The number of patients with body weight ≥60 kg also increased reaching 58 (12.9%) and 85 (21.1%) at week 22 and week 50, respectively.

**Table 1:** Characteristics of the 540 patients at ART initiation.

**Male gender**	
n (%)	351 (65)
**Age, in years**	
Median (IQR)	35 (30–41)
**Weight category, in n (%)**	
<50 kg	373 (69.1)
≥50 kg	167 (30.9)
**Weight, in kg**	
Median (IQR) all patients (n = 540)	45 (40–51)
Median (IQR) males (n = 351)	48 (44–53)
Median (IQR) females (n = 189)	39 (35–44)
**Body mass index, in kg/m^2^**	
Median (IQR) all patients (n = 540)	17.3 (15.8–19.1)
Median (IQR) males (n = 351)	18.0 (16.4–19.5)
Median (IQR) females (n = 189)	16.2 (15.1–18.2)
**CD4+ T cells, in cells/mm^3^**	
Median (IQR)	25 (12–60)
**Viral load, in log_10_ copies/mL**	
Median (IQR)	5.60 (5.20–6.0)
**Haemoglobin, in g/L**	
Median (IQR) all patients (n = 540)	101 (83–115)
Median (IQR) males (n = 351)	105 (87–120)
Median (IQR) females (n = 189)	91 (78–107)
**AST, in n (%)**	
≤1.25 ULN	249 (46.1)
1.25 to 2.50 ULN	214 (39.6)
2.50 to 5.00 ULN	55 (10.2)
>5.00 ULN	22 (4.1)
**ALT, in n (%)**	
≤1.25 ULN	487 (90.2)
1.25 to 2.50 ULN	35 (6.5)
2.50 to 5.00 ULN	15 (2.8)
>5.00 ULN	3 (0.5)
**Tuberculosis location, in n (%)**	
Pulmonary	371 (68.7)
Extra-pulmonary	51 (9.4)
Pulmonary and extra-pulmonary	118 (21.9)

ART: Antiretroviral Treatment; IQR: interquartile range; AST: aspartate aminotransferase; ALT: alanine aminotransferase.

### Plasma concentrations of efavirenz

A total of 1,759 efavirenz concentration measurements were available. Of the 540 patients, 430 (80%) had at least three efavirenz concentrations available and 401 (74.3%) had samples on and off concomitant tuberculosis treatment. A total of 40% of samples were drawn when the patients were taking rifampicin and their body weight was ≥50 kg and 8% were drawn when body weight was ≥60 kg.

**Figure 3:**
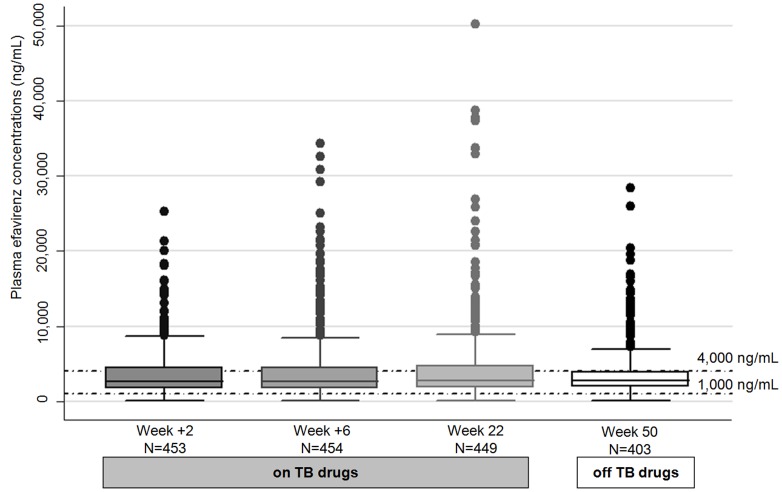
Efavirenz plasma concentrations at sampling time.

In the presence of rifampicin-based tuberculosis treatment, median [IQR] efavirenz concentrations were 2,674 ng/mL [1,690–4,533], 2,667 ng/mL [1,753–4,494] and 2,799 ng/mL [1,804–4,744] at W+2, W+6 and week 22, respectively. At week 50, six months after the end of tuberculosis treatment, efavirenz concentration was 2,766 ng/mL [1,941–3,976] (**Figure 3**). Efavirenz concentration increased between W+2 and W+6 and between W+6 and week 22, and decreased between week 22 and week 50 (p<0.001 for all three tests)

**Figure 4:**
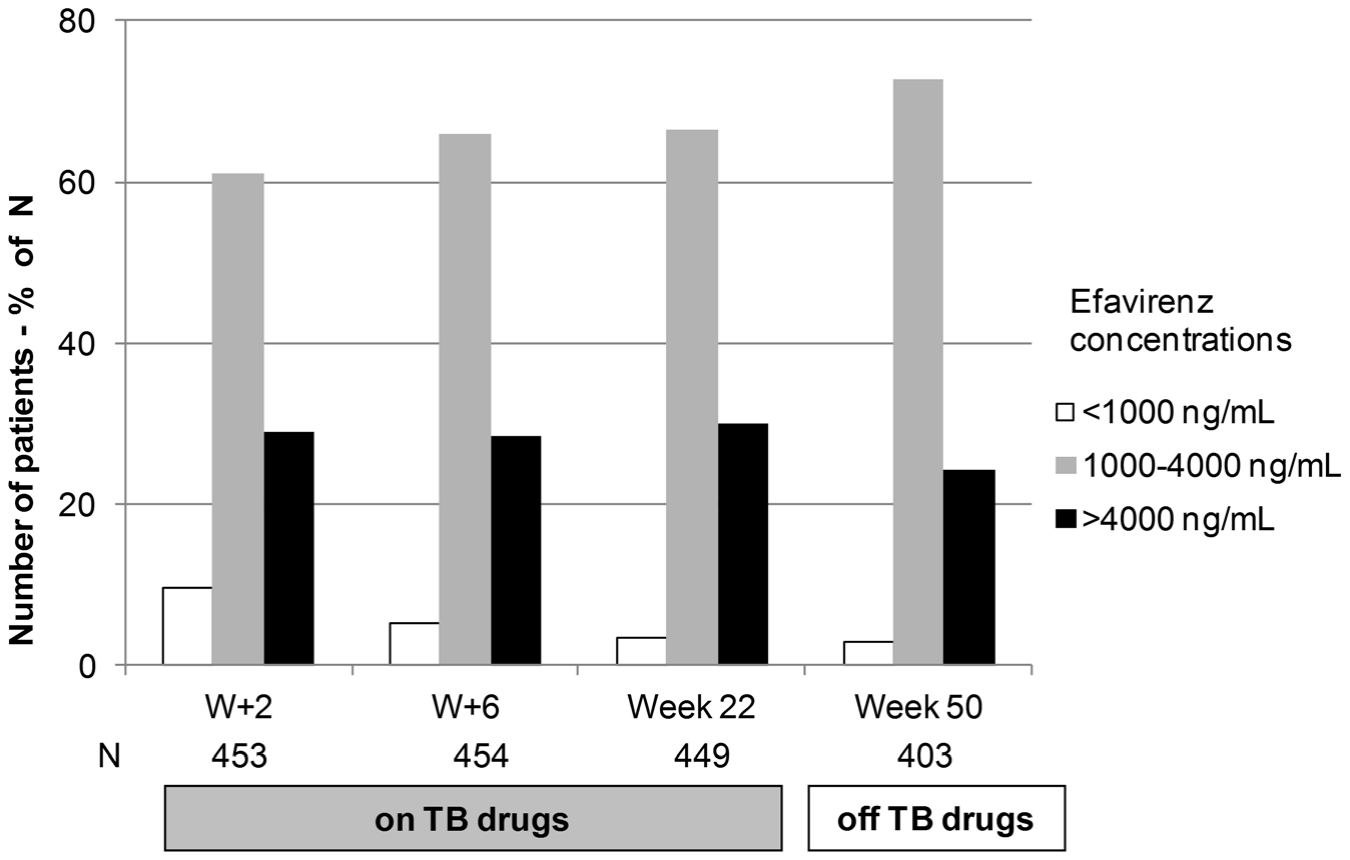
Distribution of efavirenz plasma concentrations according to the therapeutic range at each sampling time.

**Figure 5:**
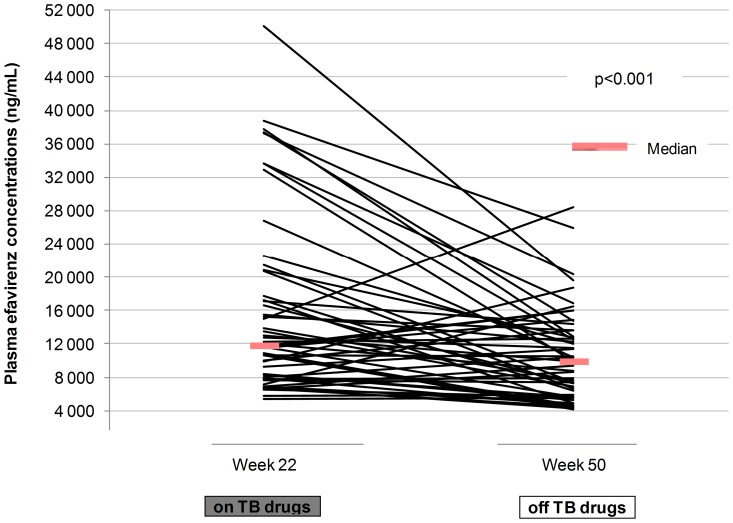
Efavirenz concentrations at week 22 and week 50 in patients whose efavirenz concentrations >4,000 ng/mL.

The distribution of efavirenz plasma concentration per sampling week is displayed in **Figure 4** **.** Efavirenz concentration was persistently within the therapeutic range in 243 patients (45.0%). At W+2, W+6 and week 22, while receiving rifampicin-based tuberculosis treatment, 44 (9.7%), 24 (5.3%) and 15 (3.3%) patients had efavirenz concentrations below 1,000 ng/mL. Off tuberculosis treatment at week 50, 12 (3.0%) patients had efavirenz concentrations below 1,000 ng/mL. The proportion of efavirenz concentrations below 1,000 ng/mL was not different on and off rifampicin-based tuberculosis treatment when comparing data collected at weeks 22 and 50 (p = 0.76). Nine (1.7%) patients had all measured concentrations below 1,000 ng/mL with an efavirenz concentration of 643 ng/mL [111–756], 829 ng/mL [828–904], and 825 ng/mL [204–915] at W+2, W+6 and week 22, respectively. These nine patients did not have efavirenz concentration available at week 50. Among them, two patients died after two and three months of follow-up, respectively, two received a non efavirenz-based regimen at week 50 and five did not have a sample drawn at this time. A total of 89 patients (16.3%) had all concentrations above 4,000 ng/mL: efavirenz concentration was 8,740 ng/mL [6,328–10,871], 10,563 ng/mL [7,068–15,106], and 11,465 ng/mL [7,639–17,748] at W+2, W+6 and week 22, respectively. In these 89 patients, there was a significantly lower efavirenz concentration at week 50 (9,633 ng/mL [5,690–13,240]) compared to the previous concentration at week 22 (p<0.001) (**Figure 5**). In multivariate analysis, factors independently associated with an increased risk of efavirenz concentrations below 1,000 ng/mL were hemoglobinemia ≤94 g/L as compared to >120 g/L (p = 0.03) and late attendance to the current protocol visit (p = 0.001). By contrast, being a woman decreased risk of efavirenz concentrations below 1,000 ng/mL (**Table 2**). Indeed, median efavirenz concentrations were higher in women compared to men at all time points (3026 versus 2468 ng/mL; 3040 versus 2441 ng/mL; 3196 versus 2665 ng/mL and 2886 versus 2737 ng/mL at W+2, W+6, weeks 22 and 50, respectively p<0.001). Notably, only 3.5% of samples drawn in women were below 1,000 ng/mL compared to 6.4% in men. Concomitant rifampicin and a body weight ≥50 kg at ART initiation and at the time of blood sampling were not associated with an increased risk of efavirenz concentration below 1,000 ng/mL.

**Table 2:** Univariate and multivariate analysis of risk factors associated with efavirenz plasma concentration below 1,000/mL.

	Number of efavirenz samples N	Efavirenz <1,000 ng/ml N (%)	Crude OR (95% CI) Univariate analysis	P	Adjusted OR (95% CI) Multivariate analysis	P
**Gender**				0.02		0.005
Male	1,163	74 (6.4)	1		1	
Female	596	21 (3.5)	0.46 (0.24–0.89)		0.37 (0.18–0.73)	
**Receiving rifampicin**				0.02		
No	385	12 (3.1)	1			
Yes	1,374	83 (6.0)	2.17 (1.11–4.21)			
**Tuberculosis location**				0.08		
Pulmonary	1,217	72 (5.9)	1			
Extra-pulmonary	167	2 (1.2)	0.16 (0.03–0.80)			
Pulmonary and extra-pulmonary	375	21 (5.6)	1.00 (0.51–1.98)			
**Weight at ART initiation**				0.06		
<50 kg	1,196	55 (4.6)	1			
50–59 kg	470	37 (7.9)	1.98 (1.08–3.64)			
≥60 kg	93	3 (3.2)	0.68 (0.15–2.99)			
**Viral load at ART initiation, log_10_ copies/mL**				0.07		
≤5	335	15 (4.5)	1			
5–6	988	66 (6.7)	1.55 (0.73–3.33)			
>6	436	14 (3.2)	0.67 (0.26–1.73)			
**Body mass index at sampling**				0.09		
≤15 kg/m^2^	118	8 (6.8)	2.08 (0.76–5.71)			
15–16 kg/m^2^	162	11 (6.8)	2.41 (1.01–2.41)			
16–17 kg/m^2^	173	8 (4.6)	1.40 (0.53–3.55)			
17–18.5 kg/m^2^	367	27 (7.4)	2.21 (1.17–4.19)			
>18.5 kg/m^2^	939	41 (4.4)	1			
**Weight at sampling**				0.04		
<50 kg	936	51 (5.4)	1			
50–59 kg	630	41 (6.5)	1.04 (0.60–1.80)			
≥60 kg	193	3 (1.5)	0.17 (0.04–0.70)			
**CD4+ T cells at sampling**						
≤50 cells/mm^3^	531	37 (7.0)	4.41 (1.70–11.42)	0.02		
51–100 cells/mm^3^	302	22 (7.3)	4.37 (1.60–11.95)			
101–200 cells/mm^3^	514	23 (4.5)	2.10 (0.81–5.44)			
201–350 cells/mm^3^	293	8 (2.7)	1			
>350 cells/mm^3^	104	4 (3.8)	1.61 (0.38–7.84)			
Missing	15	1 (6.7)	4.27 (0.31–6.84)			
**ALT at sampling**				0.14		
≤1.25 ULN	1,334	82 (6.1)	1			
>1.25 ULN	421	13 (3.1)	0.51 (0.26–1.00)			
Missing	4	0(0)	Non estimable			
**Haemoglobin at sampling**				<0.001		0.03
≤70 g/L	56	9 (16.1)	8.05 (2.70–23.99)		4.77 (1.51–15.07)	
71–80 g/L	73	9 (12.3)	6.23 (2.18–17.83)		4.06 (1.36–12.10)	
81–94 g/L	140	14 (10.0)	4.10 (1.75–9.64)		2.66 (1.08–6.58)	
95–120 g/L	612	30 (4.9)	1.68 (0.90–3.15)		1.37 (0.72–2.64)	
>120 g/L	870	32 (3.7)	1		1	
Missing	8	1 (12.5)	7.92 (0.51–121.93)		7.69 (0.57–102.99)	
**Low efavirenz concentration at previous visit**				<0.001		0.01
No	1,148	31 (2.7)	1		1	
Yes	71	13 (18.3)	2.59 (0.68–9.89)		2.04 (0.54–7.76)	
No previous sample available	540	51 (9.4)	3.70 (2.28–6.00)		2.48 (1.38–4.45)	
**Attendance at the current visit**				0.04		0.01
On time	1,727	90 (5.2)	1		1	
Late	32	5 (15.6)	4.10 (1.10–15.23)		5.36 (1.40–20.44)	
**Previous IRIS**				0.06		
No	1,354	80 (5.9)	1			
Yes	405	15 (3.7)	0.49 (0.23–1.03)			

Percentiles are gender-specific. IRIS: paradoxical tuberculosis-associated immune reconstitution inflammatory syndrome.

Sampling times were: 2 weeks after ART initiation, 6 weeks after ART initiation, at 22 weeks of follow-up, and at 50 weeks of follow-up. See text for details.

### Efficacy outcomes

At week 8 (for the 277 patients from the “early-ART” group with available viral load), week 26 and week 50, 206 (74%), 455 (91%) and 458 (97%) patients had plasma HIV RNA concentration <250 copies/mL, respectively. Patients with at least one efavirenz concentration below 1,000 ng/mL prior to the virological evaluation time did not present a lower risk of virological success at these three time-points than the other patients (median [95% confidence interval (CI)]: 0.80 [0.35–1.86], 0.31 [0.07–1.32] and 1.37 [0.38–4.94] at weeks 8, 26 and 50, respectively). Efavirenz concentrations measured in 27 and 58 patients weighing ≥60 kg at W+6 and week 22 were all above 900 ng/mL and were not associated with virological failure at week 26 (p = 1.00 and 0.89, respectively). The median [IQR] CD4+ T cells gain from baseline was 110 (69–168) cells/mm^3^ and 165 (108–226) cells/mm^3^ at W26 and W50, respectively. Similarly, having at least one efavirenz concentrations below 1,000 ng/mL prior to the immunological evaluation time was not associated with a lower CD4+ T cells gain at week 26 or week 50 (p = 0.65 and p = 0.82, respectively).

### Tolerance

During the trial, 55 (10.2%) patients experienced 55 severe (grade 3–4) adverse effects, including: 47 hepatotoxicity (85.5%); five CNS events (9.1%) -including two depressions, two deliriums and one insomnia-; three mucocutaneous events (5.4%). Overall, 23 CNS side effects of all grades were observed in 22 patients. They included sleep disorders (57%), depression (17%), vertigo (13%) and delirium (13%). One patient with severe depression committed suicide at 40.5 weeks of follow up. Efavirenz concentrations of this patient reached 13,462 ng/mL at W+6 and 25,780 ng/mL at W22.

The risk of experiencing a severe adverse effect was not significantly increased in patients who had efavirenz concentrations that was at least once above, or always above 4,000 ng/mL, when compared to patients who never had concentrations above 4,000 ng/mL (p = 0.30). However, the risk of developing a CNS side effect, irrespective of the severity, was significantly increased in patients who had efavirenz concentrations at least once above 4,000 ng/mL or those with concentrations always above 4,000 ng/mL, compared to patients who never experienced efavirenz concentrations above 4,000 ng/mL (p<0.001, OR [95% CI]: 2.72 [2.05–3.62] and p<0.001, 2.84 [2.08–3.89], respectively). The risk of developing hepatotoxicity of any grade was higher in patients with concentrations always above 4,000 ng/mL (p<0.001, OR [95% CI]: 1.52 [1.33 –1.74]). However, having intermittent efavirenz concentrations above 4,000 ng/mL did not increase the risk of hepatotoxicity and even decreased the risk (p<0.001, OR [95% CI]: 0.63 [0.56–0.70]).

## Discussion

Efavirenz plasma concentrations on and off rifampicin-containing tuberculosis treatment were documented in more than 500 Southeast Asian patients co-infected with HIV and tuberculosis from Cambodia. Our results show that after administration of a daily dose of 600 mg efavirenz, efavirenz concentrations were not decreased when efavirenz was administered with concomitant rifampicin-isoniazid containing tuberculosis treatment, irrespective of patient's body weight. A slight decrease in efavirenz concentrations was observed after tuberculosis treatment discontinuation in patients with concentrations higher than 4,000 ng/mL. Throughout the duration of the study, a total of 45% of patients had an efavirenz concentration within the 1,000–4,000 ng/mL therapeutic range. The few patients with efavirenz concentrations below the therapeutic range were not at higher risk of virological failure. Importantly, we were able to demonstrate that patients with efavirenz concentrations above 4,000 ng/mL had higher risk of CNS side effects and hepatotoxicity. This finding, in keeping with Marzolini et al.[14], had not been rigorously documented previously in resource-limited settings.

The efavirenz concentrations measured in a Cambodian population after 600 mg daily efavirenz administration, are in keeping with those reported in studies conducted mainly in Caucasian and African American or African patients or in smaller cohorts of Thai patients [18], [19]. Our data suggest that efavirenz disposition could be similar in patients from Southeast Asian countries as in patients from Sub-Saharan Africa or with African ancestors. We found that 24.3 to 30.1% of the measured efavirenz concentrations in plasma were above 4,000 ng/mL when patients received 600 mg of efavirenz daily, in agreement with other studies [20], [21]. It has been demonstrated that CYP2B6 is the main enzyme involved in efavirenz metabolism [22] and that patients carrying the loss of function *CYP2B6 516TT* genotype had a 3.5-fold increase in efavirenz exposure [18], [23], [24], [25], [26]. Given that the frequency of the loss of function allele *CYP2B6 516 T* is 34% in Cambodian population, higher than in Caucasian population, this could at least in part explain the high concentrations of efavirenz measured in our population [26], [27].

A number of studies have been conducted to assess the effect of rifampicin or rifampicin based tuberculosis treatment co-administration on efavirenz concentrations, which reported results different from those reported here. Three studies with a small number of volunteers or patients showed that plasma efavirenz levels were decreased in the presence of rifampicin alone or rifampicin-based tuberculosis treatment [27], [28], [29]. Other studies concluded that co-administration of rifampicin-based tuberculosis treatment did not decrease efavirenz concentrations [21], [30], which support our results. By contrast, recent studies demonstrated slightly increased efavirenz concentrations when co-administered with rifampicin-based tuberculosis treatment, especially in African or African-American patients [4], [20]. Our results are in agreement with these unexpected results and indicate that patients with efavirenz concentrations persistently above 4,000 ng/mL had a small decrease in efavirenz concentration at tuberculosis treatment cessation. The mechanism of this phenomenon is unclear. An inhibitory effect of isoniazid, which is always given in combination with rifampicin throughout tuberculosis treatment, has been suggested [4].

Our findings in a large patient study show no significant reduction in efavirenz concentrations with rifampicin-based tuberculosis treatment co-administration irrespective of body weight, consistent with data from a recently published study [20]. In addition, we observed a small proportion of patients with plasma concentrations below 1,000 ng/mL in our Southeast Asian population, in agreement with previous studies conducted in patients from different ethnicities [20], [21], [25], [30]. Thus, our findings strongly suggest that efavirenz dosing should not be increased when efavirenz-based ART is initiated in patients on standard tuberculosis treatment, irrespective of patient's body weight.

Of note, late attendance to protocol visits was associated with a higher risk of efavirenz concentrations below 1,000 ng/mL and may be due to drug shortage between planned and actual protocol visits. However, sustained efavirenz concentrations above 600 ng/mL have already been reported in association with its long half-life (>24 hours) [31]. Furthermore, no significant association was found between efavirenz concentrations below 1,000 ng/mL and detectable viral load at week 8 (in patients included in the “early-ART” arm), at week 26 while on tuberculosis treatment and at one year of follow-up. Overall, virological suppression was achieved in 97% of patients at week 50. In various settings, excellent virological efficacy was also observed with 600 mg efavirenz co-administered daily with rifampicin, which again supports the lack of potent drug-drug interaction between rifampicin-based standard tuberculosis treatment [32], [33], [34], [35], [36], [37], [38]. Our observation that women were at reduced risk of having lower efavirenz concentrations is in keeping with Burger *et al* [39]. Higher concentrations in women might be the consequence of lower weight compared to men. Unexpectedly, low efavirenz concentrations were related to low hemoglobinemia. The exact meaning of this finding is unclear. The majority of our patients had anaemia with median hemoglobinemia below the normal range at inclusion, which could be related to both HIV and nutritional deficiency [40]. We speculate that such advanced disease presentations could alter efavirenz absorption, which remains to be demonstrated.

High efavirenz plasma concentrations were associated with an increased occurrence of CNS side effects of all severity grades. It has been previously reported that up to 50% of patients receiving efavirenz - especially if they had a slow *CYP2B6* metabolizer genotype - were likely to experience CNS toxicity [41], [42], [43], [44], [45]. In our study, we observed a lower occurrence of CNS side effects than in those studies. No specific side effects questionnaire was administered to patients and events were collected on the basis of spontaneous information given by the patient or clinician observation during follow-up visits. It is likely that side effects were under-reported by these very advanced immunocompromised patients. Another hypothesis for this lower rate of CNS toxicity is a high tolerance for CNS side effects [14], [43], [46], [47] that were often of mild severity and decreased over time, as previously reported [41], [48].

Depending on the considered studies, efavirenz plasma concentrations above 4,000 ng/mL were [14], [43], [46], [47] or were not [24], [41], [48], [49] associated with efavirenz adverse events. In the 2NN study [50], no relationship was observed between CNS/psychiatric events and efavirenz exposure, except in Thailand (p<0.001, OR [95% CI] = 5.8 [2.5–3.6]). In this study efavirenz concentration was associated with elevation of liver enzymes in keeping with our data recorded in patients from Cambodia (p = 0.021, OR [95% CI] = 4.41: [1.3–15.5]).

Our study presents some limitations. First, patients with a high body weight were not present in our study population, as only 27 patients at W+6 and 58 patients at week 22 had a body weight of 60 kg or above. Indeed high body weight is not common in Southeast Asia and in patients with such advanced disease presentation. However, efavirenz concentrations were not associated with virological failure (observed in 8%) at week 26 in these patients. Second, efavirenz pharmacokinetic characteristics were not fully characterized in our population. Mid-dose and not pre-dose trough concentrations were collected as a surrogate of efavirenz exposure. However, such approximation is acceptable as efavirenz has an elimination half-life longer than the 24 h-dosing interval, which would minimize fluctuations between peak and trough concentrations. In addition, the genetic polymorphism of *CYP2B6 G516T*, which explained at least part of the large inter-individual variability of efavirenz concentrations, was not characterized in these 540 patients. Lastly, tolerance of efavirenz was not assessed by systematic questionnaires leading to potential under reporting of side effects. It could be argued that all effects reported in our study were representative of tolerance in real life situation. Although elevation of liver enzymes and hepatotoxicity were related to high efavirenz concentrations, efavirenz may not be the only associated factor. Tuberculosis treatment could favor transient liver toxicity and our one year study did not allow long term evaluation of efavirenz related hepatotoxicity. In addition, hepatitis C status of included patients was not recorded in the CAMELIA study. Our finding that CNS adverse effects were related to high efavirenz concentrations should favor initiation of clinical trials evaluating lower efavirenz dosing and the development of pharmacovigilance to carefully record adverse events occurrence in resource-limited countries.

In conclusion, our study provided efavirenz plasma concentrations in a large cohort of Southeast Asian patients receiving a daily standard-dose of 600 mg co-administered with rifampicin-based tuberculosis treatment. We found that efavirenz concentrations were in the same range than in other studies mostly conducted in African HIV-tuberculosis co-infected patients, demonstrating that rifampicin-based tuberculosis treatment does not impair efavirenz exposure and efficacy, irrespective of body weight. A proportion of patients with high efavirenz concentrations were at higher risk of CNS side events occurrence. Whether efavirenz dosing should be reduced in these patients to improve tolerance warrants further study.

## Supporting Information

Checklist S1 CONSORT Checklist.
(PDF)

Protocol S1 Trial Protocol.
(PDF)
