# Supplementary material for: Plasma Concentrations, Efficacy and Safety of Efavirenz in HIV-Infected Adults Treated for Tuberculosis in Cambodia (ANRS 1295-CIPRA KH001 CAMELIA Trial)
Source: PLoS One. 2014 Mar 7;9(3):e90350. doi: 10.1371/journal.pone.0090350 (PMC3946522; doi:10.1371/journal.pone.0090350)
Supplement: Protocol S1 — Trial Protocol. (PDF) [file pone.0090350.s002.pdf]

CAMELIA: Early *vs.* late introduction of antiretroviral therapy in naive HIV-infected adult patients with tuberculosis in Cambodia.

ANRS 1295

Version 6.0, November 29th, 2007

Version 5.0 : Approved by the Cambodian "National Ethic Committee for Health Research" on December 30th, 2005.

Version 4.0 August 5th, 2005 : Approved by the Cambodian "National Ethic Committee for Health Research" on August 19th, 2005.

Version 3.0 October 22nd, 2004 : Approved by the Cambodian "National Ethic Committee for Health Research" on December 3rd, 2004.

Coordinating Investigators:

Dr François-Xavier BLANC
Internal Medicine Department, Bicêtre University Hospital
78, rue du Général Leclerc
94275 Le Kremlin Bicêtre Cedex, France
Tel : +33 1 4521 2533
Fax : +33 1 4521 2632
E-mail: xavier.blanc@bct.aphp.fr

Dr SOK Thim
Cambodian Health Committee
House #64, street 592, Boeung Kak II, Tuol Kork
Phnom Penh, Cambodia
Tel: +855 12 952 858
Fax : +855 23 885 169
e-mail : sokthimcipra@online.com.kh

Dr Anne E GOLDFELD
CBR Institute for Biomedical Research
Harvard Medical School
800 Huntington Avenue
Boston MA 02115, USA
Tel : +1 617 278 3351
Fax : +1 617 278 3454

E-mail: goldfeld@cbrinstitute.org

Sponsor :

Agence Nationale de Recherches sur le SIDA et les hépatites virales
101 rue de Tolbiac
75013 Paris
Tel : +33 1 53 94 60 00
Fax : +33 1 53 94 60 02

The ANRS 1295 trial is supported by The Cambodian National Center for HIV/AIDS, Dermatology and STD (NCHADS) and the American National Institute of Allergy and Infectious Diseases (NIAID/National Institutes of Health - NIH)

CAMELIA: Early *vs.* late introduction of antiretroviral therapy in naive HIV-infected adult patients with tuberculosis in Cambodia.

ANRS 1295

Version 6.0, November 29th, 2007

Version 5.0 December 22nd, 2005 : Approved by the Cambodian "National Ethic Committee for Health Research" on December 30th, 2005.

Version 4.0 August 5th, 2005 : Approved by the Cambodian "National Ethic Committee for Health Research" on August 19th, 2005.

Version 3.0 October 22nd, 2004 : Approved by the Cambodian "National Ethic Committee for Health Research" on December 3rd, 2004.

PROTOCOL SIGNATURE

Coordinating Investigators :

François-Xavier BLANC, MD PhD
Bicêtre University Hospital
78, rue du Général Leclerc
94275 Le Kremlin Bicêtre Cedex,
France

Date :

Signature :

SOK Thim, MD
Cambodian Health Committee
House #64, street 592, Boeung Kok II,
Tuol Kork dictrict, Phnom Penh,
Cambodia

Date :

Signature :

Anne E. GOLDFELD, MD
CBR Institute for Biomedical Research
Harvard Medical School
800 Huntington Avenue
Boston MA 02115, USA

Date :

Signature :

Sponsor:

Agence Nationale de Recherches sur le SIDA et les hépatites virales
101, rue de Tolbiac
75013 Paris, France

Date :

Signature :

CAMELIA: Early *vs.* late introduction of antiretroviral therapy in naive HIV-infected adult patients with tuberculosis in Cambodia.

ANRS 1295

PROTOCOL TEAM

COORDINATING INVESTIGATORS:

**Dr François-Xavier BLANC**<br>Internal Medicine Dpt, Bicêtre University Hospital<br>78, rue du Général Leclerc<br>94275 Le Kremlin Bicêtre Cedex, France<br>Tel : +33 1 4521 2533<br>Fax : +33 1 4521 2632<br>E-mail: xavier.blanc@bct.aphp.fr	**Dr SOK Thim**<br>Cambodian Health Committee<br>House #64, street 592, Boeung Kok II, Tuol Kork<br>Phnom Penh, Cambodia<br>Tel : +855 12 952 858<br>Fax : +855 23 885 169<br>E-mail: sokthimcipra@online.com.kh
**Dr Anne E GOLDFELD**<br>CBR Institute for Biomedical Research,<br>Harvard Medical School, 800 Huntington Avenue<br>Boston MA 02115, USA<br>Tel : +1 617 278 3351<br>Fax : +1 617 278 3454<br>E-mail: goldfeld@cbrinstitute.org	

PROJECT COORDINATION:

Dr Laurence BORAND<br>Unité d'épidemiologie, Institut Pasteur<br>5 boulevard Monivong<br>Phnom Penh, Cambodia<br>Tel : +855 12 333 671<br>Fax : +855 23 428 561<br>E-mail: lborand@pasteur-kh.org

CLINICAL COORDINATION:

Dr Olivier MARCY<br>Cambodian Health Committee<br>House #64, street 592, Boeung Kok II, Tuol Kork<br>Phnom Penh, Cambodia<br>Tel : +855 12 325 925<br>Fax: +855 23 885 169<br>E-mail: oliviermarcy@yahoo.fr	Dr Didier LAUREILLARD<br>Immunologie Clinique<br>Hôpital Européen Georges Pompidou<br>20 rue Leblanc, 75015 Paris - France<br>Tel : +33 1 56 09 25 62<br>Fax : +33 1 56 09 30 26<br>E-mail: didier.laureillard@egp.aphp.fr
Dr CHAN Sarin<br>Cambodian Health Committee<br>House #64, street 592, Boeung Kok II, Tuol Kork<br>Phnom Penh, Cambodia<br>Tel : +855 12 956 674<br>Fax: +855 23 885 169<br>E-mail: sarinchan73@yahoo.fr	

METHODOLOGY COORDINATION:

Dr Sirenda VONG<br>Unité d'épidemiologie, Institut Pasteur<br>5 boulevard Monivong<br>Phnom Penh, Cambodia<br>Tel : +855 12 333 650<br>Fax : +855 23 428 561<br>E-mail: svong@pasteur-kh.org	Dr Sylvia TAYLOR<br>Unité Epidémiologie des maladies émergentes<br>Institut Pasteur, 25 rue du Docteur Roux<br>75724 Paris cedex 15, France<br>Tel : +33 1 45 68 82 17<br>Fax : +33 1 45 68 88 76<br>E-mail: sylvia.mingo@gmail.com
Dr Claire REKACEWICZ<br>Service Recherches dans les pays en développement<br>ANRS, 101 rue de Tolbiac,<br>75013 Paris, France<br>Tél : +33 1 53 94 80 91<br>Fax : +33 1 53 94 60 01<br>E-mail : claire.rekacewicz@anrs.fr	

LABORATORY COORDINATION:

Eric NERRIENET<br>Unité de Virologie, Institut Pasteur<br>5 boulevard Monivong, Phnom Penh, Cambodia<br>Tel.: + 855 12 333 105<br>Fax : +855 23 725 606<br>E-mail: enerrienet@pasteur-kh.org

ADMINISTRATIVE COORDINATION:

Dr CHIV Bunthy<br>Executive Director<br>Cambodian Health Committee<br>House #64, street 592, Boeung Kok II, Tuol Kork<br>Phnom Penh, Cambodia<br>Tel: + 855 12 952 508<br>Fax: + 855 23 885 169<br>E-mail: chc_ed@online.com.kh	Jean-Louis SARTHOU<br>Directeur de l'Institut Pasteur du Cambodge<br>Institut Pasteur du Cambodge<br>5 boulevard Monivong<br>Phnom-Penh, Cambodia<br>Tel: + 855 12 802 979<br>Fax: + 855 23 428 561<br>E-mail: sarthou@pasteur-kh.org
Jean-Paul DOUSSET<br>Directeur Administratif et Financier<br>Institut Pasteur du Cambodge<br>5 boulevard Monivong<br>Phnom-Penh, Cambodia<br>Tel: + 855 12 802 977<br>Fax: + 855 23 428 561<br>E-mail: dousset@pasteur-kh.org	

CLINICAL INVESTIGATORS ON SITES:

**KHMERO-SOVIET FRIENDSHIP HOSPITAL**<br>Dr PRAK Narom<br>Physician in Infectious disease department<br>Tel : + 855 12 893 807<br>E-mail : praknarom@yahoo.com	**CALMETTE HOSPITAL**<br>Dr HAK Chanroeurn<br>Physician in Ward B<br>Tel : + 855 92 69 75 76<br>E-mail : chanroeurn@yahoo.ca
**SVAY RIENG HOSPITAL**<br>Dr LAK Kimkhemarin<br>Chief of General medicine ward and HIV Clinic<br>Tel : + 855 12 981 018<br>E-mail : chcsr@online.com.kh	**TAKEO HOSPITAL**<br>Dr KIM Chindamony<br>Physician in Chronic Diseases Clinic<br>Tel : + 855 12 834 638<br>E-mail : cdc_takeo@camintel.com
**SIEM REAP HOSPITAL**<br>Dr CHY Say<br>Physician in Chronic Diseases Clinic<br>Tel : + 855 12 445 085<br>E-mail : MSFB-Siem-Reap@brussels.msf.org	

ANRS REPRESENTATIVES:

Dr Brigitte BAZIN<br>ANRS<br>101 rue de Tolbiac<br>75013 Paris, France<br>Tel: +33 1 53 94 60 46<br>Fax: +33 1 53 94 60 01<br>E-mail: brigitte.bazin@anrs.fr	Dr Séverine BLESSON<br>ANRS<br>101 rue de Tolbiac<br>75013 Paris, France<br>Tel: +33 1 53 94 80 95<br>Fax: +33 1 53 94 60 01<br>E-mail: severine.blesson@anrs.fr

DAIDS REPRESENTATIVES:

Ms. Jane BUPP<br>DAIDS<br>6700B Rockledge Drive<br>Bethesda, MD 20892-7624<br>Tel: +1 (301) 451-2759<br>Fax: +1 (301) 480-4582<br>E-mail: jbupp@niaid.nih.gov	Dr Lawrence FOX<br>DAIDS<br>6700B Rockledge Drive<br>Bethesda, MD 20892-7624<br>Tel: +1 (301) 402-0129<br>Fax: +1 (301) 435-9282<br>E-mail: LFOX@niaid.nih.gov

CIPRA PROJECT COORDINATORS:

Dr SOK Thim (Principal Investigator)<br>Cambodian Health Committee<br>House #64, street 592, Boeung Kok II, Tuol Kork<br>Phnom Penh, Cambodia<br>Tel : +855 12 952 858<br>Fax : +855 23 885 169<br>E-mail: sokthimcipra@online.com.kh	Dr Anne E. GOLDFELD (co-Principal Investigator)<br>CRB Institute for Biomedical Research<br>800 Huntington Avenue<br>Boston MA 02115, USA<br>Tel : +1 617 278 3351<br>Fax : +1 617 278 3454<br>E-mail: goldfeld@cbrinstitute.org

CAMELIA: Early *vs.* late introduction of antiretroviral therapy in naive HIV-infected adult patients with tuberculosis in Cambodia.

ANRS 1295

GLOSSARY

AFB	Acid-Fast Bacillus
AIDS	Acquired Immuno Deficiency Syndrome
ANRS	Agence Nationale de Recherches sur le SIDA et les hépatites virales
ART	Anti Retroviral Therapy
ARV	Anti Retroviral Drug
CBC	Complete Blood Count
CHC	Cambodian Health Committee
CRF	Case Report Forms
DAIDS	Division of AIDS
DSMB	Data and Safety Monitoring Board
FDA	Food and Drugs Administration
HAART	Highly Active Anti Retroviral Treatment
HIV	Human Immunodeficiency Virus
IPC	Institut Pasteur du Cambodge
IRIS	Immune Reconstitution Inflammatory Syndrome
MOP	Manual Of Procedures
NNRTI	Non Nucleoside Reverse Transcriptase Inhibitor
NRTI	Nucleoside Reverse Transcriptase Inhibitor
OFCP	Organisation Franco Cambodgienne de Pneumologie
OI	Opportunistic Infection
PI	Protease Inhibitor
SAB	Scientific Advisory Board
SAE	Serious Adverse Event
TB	Tuberculosis

CAMELIA: Early *vs.* late introduction of antiretroviral therapy in naive HIV-infected adult patients with tuberculosis in Cambodia.

ANRS 1295

SUMMARY

In Cambodia the prevalence of both tuberculosis (TB) and Human Immunodeficiency Virus (HIV) infection is high. In 2000, there were approximately 75.000 newly diagnosed TB cases. In 2003, 1.9% of the population was infected with HIV. TB rates in Cambodia are more than double those observed in other developing countries and up to 30 times higher than those currently seen in the USA or Western Europe. It is estimated that over 8% of the newly diagnosed TB cases are co-infected with HIV, of which approximately 85% are severely immunosuppressed (CD4+ cell count < 200 x 10^6 cells/l).

Mortality rates were found to be 2-4 folds higher in HIV/TB co-infected patients than in TB alone. Data suggest that aggressive management of HIV infection, which includes Highly Active Anti-Retroviral Therapy (HAART) during treatment of TB decreases both morbidity and mortality by suppressing viral replication and improving immune function.

On the other hand, the use of HAART for patients with TB may cause severe complications due to drug-drug interactions, and occasionally a temporary exacerbation of symptoms, signs or radiographic manifestations of TB. Such events or 'paradoxical reactions' that occur among 7 - 36% of HIV/TB co-infected patients treated with HAART may be secondary to immune restitution. These reactions may be particularly severe when HAART is started soon after the start of TB treatment.

Most clinical teams recommend delaying the initiation of HAART to avoid the early side effects of TB treatment and simplify clinical management of the co-infected patient. However others argue that early initiation of HAART in TB patients with CD4 cell counts < 100 x 10^6 cells/l leads to a marked reduction of viral load despite frequent adverse events.

The proposed study aims to determine the optimal time to initiate HAART (defined as d4T + 3TC + efavirenz) in previously untreated HIV-infected adult patients with TB and low CD4 cell counts. The study is a multicentre prospective, randomized, open-label two-armed trial with no placebo. It is designed as a superiority trial to compare the “early arm” (HAART initiated 2 weeks after TB treatment onset) with the "late arm" (HAART initiated 2 months after TB treatment onset). Efficacy will be assessed by the survival rate at the end of the trial, which is extended until one year after the last patient enrollment. Secondary objectives will be: 1.To compare survival rate 50 weeks after enrollment; 2. In case of TB recurrence, to differentiate TB relapse vs. TB reinfection; 3.To evaluate the safety of an early initiation of HAART in terms of side effects, drug-drug interactions, TB paradoxical reactions and IRIS; 4.To evaluate the occurrence of OI; 5.To evaluate the rate of hospitalization for any cause; 6. To measure the effectiveness of TB treatment; 7.To evaluate the occurrence of TB recurrence; 8.To measure the effectiveness of HAART (immunological restoration, rate of patients with undetectable viral load); 9.To determine predictive factors for the survival, the response to TB treatment and HAART and the paradoxical reactions (i.e. clinical, biological); 10.To evaluate patients' adherence to TB treatment and HAART; 11.To evaluate efavirenz exposure in plasma and detect any potent drug-drug interaction between efavirenz and rifampicine.

The total study duration is expected to be 4 years (3 years for enrollment, one year of follow-up) in five study sites: (1) Khmero-Soviet Friendship Hospital (formerly known as Preah Bath Norodom Sihanouk Hospital), Phnom Penh; (2) Calmette Hospital, Phnom Penh; (3) Provincial Hospital,

Svay Rieng province; (4) Provincial Hospital, Takeo province; and (5) Provincial Hospital, Siem Reap province.

The study will be carried out in compliance with the protocol and in accordance with the Declaration of Helsinki approved by the World Health Association and with the recommendations of the Good Clinical Practice.

CAMELIA: Early *vs.* late introduction of antiretroviral therapy in naive HIV-infected adult patients with tuberculosis in Cambodia.

ANRS 1295

TABLE OF CONTENTS

PROTOCOL SIGNATURE 2
PROTOCOL TEAM 3
GLOSSARY 6
SUMMARY 7
TABLE OF CONTENTS 9

1 RATIONALE 11

2 STUDY OBJECTIVES 12

2.1 PRIMARY OBJECTIVE 12
2.2 SECONDARY OBJECTIVES 13
2.2.1 New ones 13
2.2.2 Previous ones 13

3 STUDY DESIGN 13

4 ELIGIBILITY CRITERIA 14

4.1 INCLUSION CRITERIA 14
4.2 NON INCLUSION CRITERIA 15

5 TRIAL TREATMENTS 15

5.1 TREATMENT DESCRIPTION 15
5.1.1 TB treatment 16
5.1.2 Antiretroviral treatment 16
5.1.3 Associated drugs 17
5.2 HANDLING OF STUDY MEDICATION 17

6 ENDPOINTS /OUTCOMES 18

6.1 PRIMARY ENDPOINT 18
6.2 SECONDARY ENDPOINTS 18
6.2.1 Survival rate 50 weeks after enrollment 18
6.2.2 Evaluation of safety 18
6.2.3 Immune Reconstitution Inflammatory Syndrome 18
6.2.4 Paradoxical reactions 19
6.2.5 Occurrence of opportunistic infections 19
6.2.6 Evaluation of TB treatment 19
6.2.7 TB recurrence 19
6.2.8 Evaluation of ART 19
6.2.9 Resistance 20
6.2.10 Adherence 20
6.2.11 Pharmacokinetic study 20

7 TRIAL PROCEDURES 20

7.1 SCREENING 20
7.2 ENROLLMENT 21
7.3 PATIENT FOLLOW-UP 21
7.3.1 Patients' schedule 21
7.3.2 Pharmacokinetics 22
7.3.3 Adverse Event 22
7.3.4 TB treatment failure or TB relapse 23
7.3.5 ARV treatment failure 23
7.3.6 Trial or treatment premature discontinuation and loss to follow-up 23
7.4 FROZEN SAMPLES 23

8 STATISTICAL CONSIDERATION 23
8.1 SAMPLE SIZE CALCULATION 23
8.2 RANDOMIZATION PROCEDURES 24
8.3 INTERMEDIATE ANALYSIS 24
8.4 ANALYSIS PLAN 24
9 MONITORING AND DATA MANAGEMENT 25
9.1 MONITORING 25
9.1.1 On site 25
9.1.2 Coordinating centre 26
9.2 DATA MANAGEMENT 26
9.2.1 Data collection 26
9.2.2 Data entry and checking 27
9.3 TRIAL DOCUMENTATION STORAGE 27
10 TRIAL OVERSIGHT 27
10.1 OPERATIONAL TEAM 27
10.2 SCIENTIFIC ADVISORY BOARD (SAB) 27
10.3 DATA SAFETY MONITORING BOARD (DSMB) 27
11 ETHICS AND REGULATORY CONSIDERATIONS 28
11.1 ETHICS COMMITTEE AND HEALTH AUTHORITIES 28
11.2 AMENDMENTS TO THE PROTOCOL 28
11.3 DISCLOSURE AND CONFIDENTIALITY 28
11.4 INSURANCE 29
11.5 PARTICIPANT BENEFITS AND RISKS 29
11.5.1 Benefits 29
11.5.2 Risks 29
12 PUBLICATIONS 29
13 REFERENCES 31
14 ANNEXES 33
ANNEX 1: STUDY SITES 33
ANNEX 2: WORLD HEALTH ORGANIZATION STAGING SYSTEM FOR HIV INFECTION AND DISEASE 35
ANNEX 3: PATIENTS' SCHEDULE 36
ANNEX 4: INFORMATION SHEET FOR PATIENTS AND CONSENT FORM 37
ANNEX 5: HELSINKI DECLARATION 46
ANNEX 6: ETHIC COMMITTEE APPROVAL AND MINISTRY OF HEALTH AUTHORIZATION 49
ANNEX 7: INSURANCE 52

CAMELIA: Early *vs.* late introduction of antiretroviral therapy in naive HIV-infected adult patients with tuberculosis in Cambodia.

ANRS 1295

1 Rationale

Human Immunodeficiency Virus (HIV)/Acquired Immune Deficiency Syndrome (AIDS) is the modern world's principal pandemic with over 42 million people currently infected with HIV worldwide (1). Despite a significant decrease during the last years Cambodian HIV prevalence remains the highest in South East Asia with 1.9% of the population infected with HIV (157 000 persons between 15-49 years old living with HIV) (2).

Tuberculosis (TB) remains an important problem in HIV-infected patients (3). In 1997, WHO estimated that 8 million new cases of TB with 8% of new TB cases co-infected with HIV globally (4). It is the major opportunistic infection (OI) among HIV-infected patients and the major cause of death in the setting of AIDS. Cambodia is among the highest TB incidence countries worldwide. In 2000, there were 75.000 newly diagnosed TB cases. TB rates in Cambodia are more than double those observed in other developing countries and up to 30 times higher than those currently seen in the USA or Western Europe. In 2002, 8.4% of patients with active TB were co-infected with HIV. A recent study on etiologies of pulmonary disease in HIV-infected people found that more than 85% patients with pulmonary TB are severely immunosuppressed (CD4+ cell count < 200 x 10^6 cells/l) (Chan Sarin et al, 35th IUATLD World Conference, Paris 2004).

In TB/HIV co-infected patients the risk of death is much higher than patients without HIV (5), even when the organism is susceptible to the TB drugs used and response to therapy is positive (6). Both in the US and Kenya, mortality rates were found to be 2 to 4 folds higher in HIV/TB co-infected patients than in TB alone (7, 8). In Cambodia a death rate of approximately 30% was found among patients with HIV/TB co-infection (9, 10).

The degree of immunosuppression is the most important predictor of survival in HIV-infected patients with TB (3, 11). It has been shown that *Mycobacterium tuberculosis* increases HIV replication both *in vivo* and *in vitro* models (12, 13). Hence, data (14-17) suggest that aggressive management of HIV infection, which includes antiretroviral treatment (ART) during TB treatment, might improve the survival by suppressing viral replication and improving immune function (11).

Highly Active Anti-Retroviral Therapy (HAART) has been shown to decrease both HIV-related morbidity and mortality (18, 19). Such potent ART usually involves three-drug regimens with two nucleoside reverse transcriptase inhibitors (NRTI) in association with one protease inhibitor (PI) or one non-nucleoside reverse transcriptase inhibitors (NNRTI).

On the other hand, the use of HAART for patients with TB is complicated because of drug interactions between both PI and NNRTIs and rifamycin types, which together with isoniazid are critical in TB therapy. Some of these drug-drug interactions are so dramatic that they are strong contra-indications to the concurrent use of certain rifamycins and antiretroviral drugs (20, 21). In addition, the reconstitution of immune function because of HAART can also result in a transient worsening or appearance of new signs, symptoms, or radiographic manifestations of TB (22). Such events or 'paradoxical reactions' may be secondary to restoration of immunity toward mycobacterial antigens (23) including restored proliferation of T cells and elaboration of proinflammatory cytokines in response to TB antigens (24). It can occur among 7 to 36% of HIV/TB co-infected patients treated with HAART (22, 25, 26). These reactions may be particularly severe when HAART is started soon after the start of TB treatment (27).

The presence of overlapping toxicity profiles of some anti-TB and HAART drug interactions and the possibility of paradoxical reactions has led to the idea that delaying the start of HAART until there has been time to manage the early side effects of TB treatment would simplify clinical management of the co-infected patient (20). For example, one recommendation put forward has been to delay ART until the first two months of treatment for TB have been completed, even among patients who have low CD4 cell counts (20, 27).

By contrast, based on data of an observational retrospective study, Dean and colleagues recommend early initiation of HAART in TB patients with CD4 cell counts < 100 x 10^6 cells/l and argue for HIV treatment two weeks after the initiation of TB treatment (28). Preliminary results of a Brazilian phase IV, open-label, non-controlled trial support early initiation of HAART in patients receiving TB treatment and indicate that the introduction of two nucleoside analogues plus ritonavir/saquinavir one month after the TB treatment onset (including rifampicin) leads to marked reduction of viral load despite frequent adverse events (29).

In Cambodia, HAART has been available since 2001 for people living with HIV/AIDS with CD4 count less than 200 x 10^6 cells/l. The 2007 revision of the Cambodian guidelines that were originally written in December 2003 recommends d4T + 3TC + nevirapine as the first line ART, now for patients with lymphocytes T $CD4^+$ less than 250 x 10^6 cells/l instead of less than 200 x 10^6 cells/l (30). For TB/HIV co-infected patients, it is recommended to use efavirenz instead of nevirapine because of the interaction between nevirapine and rifampicine (30).

The aim of the present study is to determine the optimal time to initiate HAART (defined as d4T + 3TC + efavirenz) in previously untreated HIV-infected adult patients with TB and low CD4 cell counts. The question remains an extremely important clinical issue to be clarified and must address the risk of having to discontinue therapies because of toxicity, including side effects of multiple drugs, drug-drug interactions and the development of paradoxical reactions versus the risk of HIV-1 disease progression and mortality because of a prolonged period of low CD4 cell counts and concomitant severe immunocompromise. The underlying hypothesis is that an early initiation of HAART will increase survival in HIV/TB co-infected patients, despite a more complex initial management.

The results of this study will be of particular importance in resource-poor countries with a high prevalence of TB because of the profound level of immunosuppression usually found in HIV-1 infected individuals at the time when TB is diagnosed.

The present proposal named CAMELIA is a co-partnership between the Cambodian National Center for HIV/AIDS, Dermatology and STD (NCHADS), the French “Agence Nationale pour les Recherches contre le SIDA et les hépatites virales” (ANRS) and the American National Institutes of Health (NIH).

Since version 5.0 of the CAMELIA trial protocol, the first patient was enrolled on 31st of January 2006 and the Data and Safety Monitoring Board of the trial has met 3 times. During their last meeting in Paris on 9th of May 2007, they recommended extension of the duration of follow-up for each patient until the calendar date end of the study.

2 Study objectives

2.1 Primary objective

To compare the patients’ survival at the end of the trial (50 weeks after last patient enrollment) in an early HAART initiation arm (2 weeks after TB treatment onset) versus a late HAART initiation arm (2 months after TB treatment onset), considered as the reference arm, in drug naive HIV-infected adult patients with newly diagnosed TB.

2.2 Secondary objectives

2.2.1 New ones

These secondary objectives did not exist as secondary objectives in version 5.0 of the CAMELIA trial protocol and have been added.

1. Compare survival rate 50 weeks after enrollment
2. In case of TB recurrence, differentiate TB relapse vs. TB reinfection

2.2.2 Previous ones

These objectives already existed in version 5.0 of the CAMELIA trial protocol:

1. Evaluate the safety of an early initiation of HAART in terms of drug interactions, paradoxical reactions or IRIS
2. Evaluate the occurrence of OI
3. Evaluate the rate of hospitalization for any cause
4. Measure the effectiveness of TB treatment
5. Evaluate the occurrence of TB recurrence
6. Measure the effectiveness of HAART (immunological restoration, rate of patients with undetectable viral load)
7. Determine predictive factors for the survival, the response to TB treatment and HAART and the paradoxical reactions (i.e. clinical, biological)
8. Evaluate patients' adherence to TB treatment and HAART
9. Evaluate efavirenz exposure in plasma and detect any potent drug-drug interaction between efavirenz and rifampicine

3 Study design

This is a multicentre prospective, randomized, open-label two-armed trial with no placebo. The trial is designed as a superiority trial to answer the question of the timing for the introduction of the HAART (early vs. late introduction) in HIV-infected adult patients with TB in Cambodia.

The total study duration is expected to be four years. Each patient will be followed until the last included patient is at week 50 after initiation of TB therapy.

Study sites are described in Annex 1 and listed above:

- Khmero-Soviet Friendship Hospital in Phnom Penh
- Provincial Hospital in Svay Rieng
- Calmette Hospital in Phnom Penh
- Provincial Hospital in Takeo
- Provincial Hospital in Siem Reap

The total number of subjects to be enrolled in the trial is 660 (330 in each treatment arm).

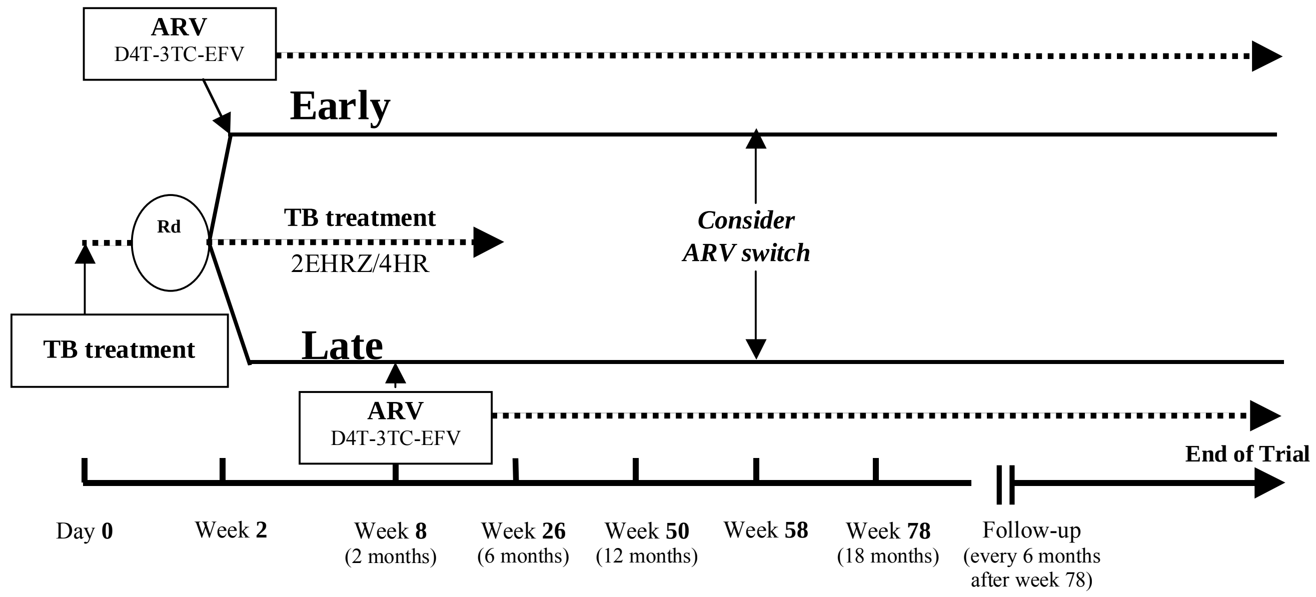

RdŹ:Randomization
HŹ:isoniazid ZŹ pyrazinamide
RŹ rifampicin EŹ ethambutol
D4T : stavudine
3TC : lamivudine
EFV : efavirenz

CAMELIA participants may be asked to volunteer for other scientific studies. These studies include but are not limited to: 1) a study focusing on the relationship between efavirenz pharmacokinetics and a genetic polymorphism influencing an enzyme involved in metabolism of efavirenz (PECAN ANRS 12154 study) and/or 2) two different studies defining the role of different cells in the phenomenon of paradoxical reactions (CAPRI NK ANRS 12153 study, CAPRI T ANRS 12164 study). Participation in any of these scientific studies will be proposed by the team on site after an explanation of the goals of each study. These studies will also enroll patients not enrolled in the CAMELIA and this in accordance with the rules and approval of the Cambodian National Ethical Committee. Medical care and CAMELIA participation will not be affected by any refusal to any of these scientific studies. Participation to any of these scientific studies is absolutely not required for participation in the CAMELIA study. The performance of any of these scientific studies will be carried out in a manner that will not produce any negative impact upon the CAMELIA study.

4 Eligibility criteria

4.1 Inclusion criteria

Male and female subjects with a diagnosis of HIV-infection and a pulmonary or extra-pulmonary TB meeting all criteria listed below:

1. Age 18 or over. No upper age limit will be applied,
2. Positive HIV test result[1],
3. CD4+ cell count ≤ 200 x 10^6 cells/l within 14 days prior the study entry
4. Positive AFB on any smear (sputum, lymph node drainage, stool, CSF, pleural fluid)

[1] The HIV tests are those used in the national Voluntary Counselling and Testing Centres according to the national guidelines which recommend two rapid HIV tests. The main tests used are: Determine, Unigold and Serodia. In all cases, HIV infection will be confirmed by the HIV viral load done on the first blood collection of the study.

5. Naive to ART
6. TB treatment started less than one week prior enrollment
7. Negative gonadotrophin pregnancy test (blood) for women of childbearing potential (i.e. not surgically sterile or <2 years menopause).
8. Agreement from female candidates who are participating in sexual activity that could lead to pregnancy while receiving and for 6 weeks after stopping efavirenz to use two reliable methods of contraception, one of which including condom.

4.2 Non Inclusion criteria

Subjects presenting with any of the following will not be included in the study:

1. Age < 18
2. Negative HIV test result
3. CD4+ cell count > 200 x 10^6 cells/l
4. Suspected TB with negative AFB
5. Pregnant or breastfeeding women
6. Impaired hepatic function (icterus, elevated AST or ALT at least 5 times over the normal value)
7. Unable and/or unlikely to comprehend and/or be adherent to the protocol
8. Treated for a previous suspected or documented TB other than the ongoing infection which motivates enrollment in this trial
9. Previous ART

Patients presenting an OI are eligible for the trial. OIs will be concomitantly treated throughout the trial.

5 Trial treatments

5.1 Treatment description

Participants will receive standard TB treatment. With respect to the introduction of HAART, participants will be randomly assigned to the "early arm" (2 weeks ± 4 days after TB treatment onset) and the "late arm" (2 months ± 4 days after TB treatment onset). ARVs are considered as the study drugs.

**Early arm**	Day 0	Initiation of TB treatment and pre-inclusion analysis
	Day 1-4	Enrollment and randomization
	Week 2*	Initiation of HAART
	Week 26	End of TB treatment
**Late arm**	Day 0	Initiation of TB treatment and pre-inclusion analysis
	Day 1-4	Enrollment and randomization
	Week 8	Initiation of HAART
	Week 26	End of TB treatment

* week# indicates the last day of the week/month

5.1.1 TB treatment

All participants will start anti-TB treatment as soon as TB is documented (AFB+). TB treatment will be administered according to the guidelines of the Cambodian National Centre for TB and Leprosy Centre. The standard 6 month-course regimen is divided into two phases. It includes rifampicin, isoniazid, ethambutol and pyrazinamide for the first two months (1st phase of the TB treatment), and isoniazid and rifampicin for the last four months (2nd phase of the TB treatment).

First two months	Rifampicin	10 mg/kg/day
	Isoniazid	4-5 mg/kg/day
	Ethambutol	15-20 mg/kg/day
	Pyrazinamide	20-30 mg/kg/day
Last four months	Rifampicin	10 mg/kg/day
	Isoniazid	4-5 mg/kg/day

In addition, Pyridoxine (10 mg-25 mg Once Daily) will be given as a supplement during the total duration of isoniazid.

Clinicians will be allowed to prolong TB treatment depending on TB localisation and results of drug susceptibility test, as indicated in the Manual of Procedures (MOP).

Given that there will be a longer duration of follow-up for most of the patients (>50 weeks), we reasonably expect that we will observe TB recurrence. During this extended follow-up, when TB recurrence is detected, TB treatment will be started immediately. Therefore, the choice of the regimen used before DST is available will depend on the DST of the first strain isolated on the specific patient. If the strain is sensitive to streptomycin, it will be added during the first 2 months, as is recommended by the National TB Program. When DST of the strain that caused the newly detected TB recurrence is available, the TB regimen will be modified and adapted to the result of DST (e.g. stop Streptomycin and give 4 months of isoniazid-rifampin when the TB strain is multi-sensitive).

5.1.2 Antiretroviral treatment

All antiretroviral drugs used in CAMELIA are in accordance with the current national ART guidelines approved by Cambodian Ministry of Health and either pre-qualified by the WHO or with tentative approval from the United States (US) Food and Drug Administration (FDA). Where feasible, generic formulations are provided to the patients. Priority is given to fixed dose combinations (FDC). DAIDS will need to agree on the use of any first line, alternative or second line drugs that are not approved by the US FDA. Fixed dose combinations of antiretrovirals or other single formulation generic drugs may be used as they become available and are agreed upon or are tentatively approved by the US FDA.

Due to the longer duration of follow-up in some patients (greater than 50 weeks), we will distinguish two phases regarding HAART.

First phase of ART: The first line regimen of ART associates D4T (Stavudine, NRTI), 3TC (Lamivudine, NRTI) and efavirenz (NNRTI) and will be provided until week 50 as proposed in the version 5.0 protocol.

**D4T/3TC**	D4T 30 mg/3TC 150 mg, 1 tablet 2 times per day
**Efavirenz**	1 tablet 600 mg Once a Day (OD)

However, some patients will have already been switched to another combination due to side effects or therapeutic failure before week 50. At week 50, each patient will be given 2 extra months of his/her ongoing HAART regimen. During these 2 extra months, onsite physicians will gather CD4, viral load and genotyping when viral load is detectable so that they will be in the optimal position to decide upon the HAART regimen for the following months.

Since March 2005, efavirenz is classified as a FDA Class D or teratogenic drug and can not be used during pregnancy. Pregnancy test will be repeated as needed for clinical suspicion of pregnancy.

Second phase of ART: During this phase, patients will receive ARVs from the Cambodian National Centre for HIV/AIDS, Dermatology and STD (NCHADS). The first visit of this phase of ART will occur at week 58. At this time, no regimen will be imposed and the choice of HAART regimen will be under the sole responsibility of the site physicians, according to the Cambodian guidelines. Some recommendations will be given to help site physicians to consider a combination with fewer potential long term side effects (e.g., D4T switched to AZT to minimize the risk of mitochondrial toxicities and efavirenz switched to nevirapine to decrease the risk of long-term neurological and metabolic toxicities, reduce costs and, for women, avoid the risk of fetal injury in case of pregnancy).

Management of ART: Management of concomitant medications with ART is detailed in the MOP.

In case of serious side effect, ART will be changed as detailed in the MOP, according to international recommendations and the National Cambodian Guideline for the use of Anti-Retroviral Treatment (ART) in adults and adolescents.

In the case of treatment failure, a second line treatment will be given according to international recommendations and the National Cambodian Guideline for the use of Anti-Retroviral Treatment (ART) in adults and adolescents.

5.1.3 Associated drugs

During follow-up, active prophylaxis of OI will be given using:

- Cotrimoxazole 960 mg OD (*Pneumocystis jiroveci* pneumonia prophylaxis). Discontinuation of cotrimoxazole when CD4 > 200 x 10^6 cells/l during 6 consecutive months.
- Fluconazole 100-200 mg OD when CD4 < 100 x 10^6 cells/l (*Cryptococcocus neoformans* prophylaxis). Discontinuation of fluconazole when CD4 > 100 x 10^6 cells/l during 6 consecutive months.

In case of OI treatment will be provided during the trial according to international recommendations and the National Cambodian guideline for the clinical management of HIV infection in adult (December 1998).

5.2 Handling of study medication

During the first study phase, ARVs, antiTB drugs, fluconazole and cotrimoxazole will be purchased by the ANRS and transported to the main office of Cambodian Health Committee (CHC) in Phnom Penh. A licensed Pharmacist will be responsible for drug management and shipping to the study sites where the dispensation to the patient will be done.

Drugs will be openly labeled. No specific packaging will be designed for the study. Treatment management and dispensation will be monitored according to Good Clinical Practice (ICH-E6 step 4 -1996).

After Week 58, ARVs will be purchased, managed and dispensed by each site pharmacy.

6 Endpoints /outcomes

6.1 Primary endpoint

The primary endpoint is survival at the end of the study. As the vital status will now be tracked until the end of the trial, the overall survival curves between the two arms of the study will be estimated and compared. Survival curves at the end of the trial will be compared using the log rank test.

6.2 Secondary endpoints

6.2.1 Survival rate 50 weeks after enrollment

6.2.2 Evaluation of safety

Safety will be analyzed according to type, frequency and severity of Adverse Events that occur during the trial and to their potential relations with the drugs, HIV or TB infection. An adverse event is defined as the occurrence of any harmful event (clinical or biological) among trial participants, whether or not the event is related to the study drug(s).

A **Serious Adverse Event** (SAE) (ICH-E6 step 4 - 1996) is any untoward medical occurrence that at any dose:
- results in death,
- is life-threatening,
- requires inpatient hospitalization or prolongation of existing hospitalization,
- results in persistent or significant disability/incapacity,
- is a congenital anomaly/birth defect.

All adverse events considered serious by the investigator should be reported by the investigators on site to the coordinating centre at the Pasteur Institute in Cambodia (IPC) according to the last updated methodology site MOP. The monitors together with the clinical coordinators will review the cases for completeness and send the report forms to the ANRS and DAIDS RCC Safety Office.

Modified version of ANRS "SAE report form" and modified "Procedure for serious adverse events reporting for ANRS sponsored research in developing countries" (April 2004) will be used. The modifications done on these documents were specifically made for the CAMELIA trial in order to comply with both ANRS and DAIDS requirements. ANRS will send safety report (line listing and copy of the report forms) every 6 months to DAIDS Regulatory Compliance Center (RCC) Safety Office. Events will be coded using the Medical Dictionary for Drug Regulatory Affairs (MedDRA), an international terminology developed under the auspices of the International Conference on Harmonization (ICH). The event will be graded using the "Division of AIDS table for grading the severity of adult and pediatric adverse events" (Version 1.0 – December 2004). All grade 4 events according to the grading table will be considered as SAE for this protocol and should be reported.

All adverse events should be reported including those which are not considered as potentially serious by the investigator.

6.2.3 Immune Reconstitution Inflammatory Syndrome

The Immune Reconstitution Inflammatory Syndrome (IRIS) is defined as:

- Symptoms and/or signs that are consistent with an infectious/inflammatory condition occurring at any time after initiation of HAART.

AND

- Evidence of an increase in CD4+ cell count and/or decrease in HIV-1 viral load.

AND

- These symptoms and/or signs can not be explained by a newly acquired infection, the expected clinical course of a previously recognized infectious agent, or the side effects of ART itself.

6.2.4 Paradoxical reactions

TB paradoxical reaction is defined as worsening or emergence of signs or symptoms of TB (*e.g.* fever, cough, shortness of breath, adenopathy or exacerbation of disease at other extra pulmonary sites) during appropriate TB treatment to which the patient has initially responded. Clinical worsening within evidence of nonadherence to treatment will not be considered as paradoxical reaction. TB paradoxical reaction can occur in patients before or after initiation of HAART.

6.2.5 Occurrence of opportunistic infections

All **OI** will be defined according to the WHO classification (Annex 3).

6.2.6 Evaluation of TB treatment

The treatment outcome will be defined as:

Cured:
A patient will be considered as cured at the end of the TB treatment according to the following:
- pulmonary TB: clinical and radiological improvement + negative smear test
- extra-pulmonary TB: clinical improvement

Failure:

A patient **on appropriate TB treatment** will be considered as treatment failure if there is evidence of active TB:
- when pulmonary TB sputum smears remain positive after 6 months of TB treatment
- when extra-pulmonary TB patients show no clinical improvement

Died: death occurring during the TB treatment phase

Treatment interrupted: treatment interruption for 2 months or more

Loss to follow-up: patient who failed to attend the 6-month follow-up visit (end of anti TB treatment)

6.2.7 TB recurrence

TB recurrence is defined as a new AFB positive smear after the patient has completed TB treatment and has been considered cured. TB recurrence will be determined in all patients during follow-up visits by careful physical examination. When suspected, TB will be checked by systematic examination of sputum or any other sample (e.g. stools, adenopathy…).

To investigate mechanisms of confirmed TB recurrence, molecular typing of TB strains by spoligotyping or Mycobacterial Interspersed Repetitive Units-variable number of tandem repeats (MIRU-VNTR) will be used to distinguish between reinfection and relapse as the cause of TB recurrence.

6.2.8 Evaluation of ART

Effectiveness of HAART will be assessed by analyzing the number of patients with undetectable viral load and by increases in CD4 T cell count. The success of ART here is defined as an increase

of CD4 > 50 x 10^6 cells/l above baseline and undetectable HIV plasma viral load measured by real time RT PCR for HIV-1 RNA plasmatic quantification (< 400 copies/ml).

6.2.9 Resistance

Resistance to ARV treatment will be determined by genotyping HIV-1 strains among patients with detectable viral load on Day 0 and Week 50.

Resistance to TB treatment will be determined by performing a drug-susceptibility test on the initial AFB positive sample and, for pulmonary TB, on week 8 and 26 control sputum if culture still positive at these times.

6.2.10 Adherence

Patient adherence to TB and ARV treatment will be evaluated based on interviews and pill counts at each study visit.

6.2.11 Pharmacokinetic study

To assess efavirenz plasma exposure and to detect any major decrease in efavirenz concentrations during rifampicin coadministration, efavirenz plasma concentration will be assayed at regular time intervals (see §7.3.2).

7 Trial procedures

7.1 Screening

All patients newly diagnosed with positive AFB smear will receive TB treatment immediately and be tested for HIV after HIV pre-testing counselling. The TB diagnosis and HIV testing will be done on site according to national procedures. Training and quality control for laboratory testing will be organized before the beginning of the trial.

For patients with positive AFB smear and positive HIV serology, the **investigator on site** could propose the participation to the trial after verification of the following criteria: (1) age eligibility (2) no evidence of pregnancy (3) no previous TB treatment or ART.

Patient participation in the clinical trial is voluntary, and each participant will be informed individually by the **clinical monitor** of the purpose, scope of the study procedures involved, the expected duration, the potential risks and benefits involved and any discomfort it may entail. Each participant and the clinical monitor will then sign the information and consent form prior to participation (Annex 4) and be assigned a unique ID number. It will be emphasized that participant will only be able to enter the trial if subsequently the clinical and biological criteria are met (e.g. CD4 cells count is < 200 x 10^6 cells/l).

A copy of the information sheet and the signed consent form which are written in Khmer language will be given to each participant.

When the inform consent sheet is signed, eligibility criteria will be checked by:

- Interview and clinical examination
- Blood sample will be send to IPC for Complete Blood Count (CBC), CD4 cell counts, liver function tests, biochemistry, pregnancy test for women and HIV viral load. Frozen plasma will be stored.

The interview and clinical exam will look for general health status and symptoms indicative of OI (e.g. headache, diarrhea, ascitis …). A chest X-ray will be performed if a recent one (within 15 days) is not available in the patient's record.

The presence of a concomitant OI is not an exclusion criterion for the trial and will be treated as needed.

If the inclusion criteria are not met, the patient will be followed in the same facility according to each centre procedures.

A record of all screened patient will be kept on each site, and the reason of non enrollment reported.

7.2 Enrollment

Results of the screening tests (CBC, CD4 cell counts, transaminases and pregnancy test) should all be returned to the study site within a week of TB treatment onset. Once the inclusion criteria are met, the **investigator** of each site will make the final enrollment decision and request the random allocation to one of the two arms from the epidemiology unit at the IPC. The allocation should be done within a week after the beginning of the TB treatment. The time between the onset of TB treatment (D0) and the random allocation is considered as the screening period. The baseline characteristics and the first blood collection will be done during the screening period (7 days at most).

In order to be consistent and simplify the planning of each patient treatment schedule, we will call “Day 0” (D0) the date of onset of the TB treatment.

Participants will be treated either as in-patients or as out-patients depending on the level of their medical needs at the time of initial evaluation.

7.3 Patient follow-up

7.3.1 Patients’ schedule

When enrolled in the study, participants will be clinically and biologically monitored on a regular basis as described in the “Patients’ schedule” table (Annex 3). Study visits are scheduled two weeks after TB treatment onset, two weeks after the initiation of ARV, every four weeks during the TB treatment phase, every two months until week 58, at week 78 then every six months until the end of the study. An allowance of ± 2 days around each protocol visit will be given.

At onset of ART potential childbearing women will be retested for pregnancy (urinary test).

At each protocol visit, patients will be interviewed and examined for potential side-effects, evaluated for clinical efficacy of anti-TB treatment and ART and will receive laboratory testing as indicated in the patients’ schedule table (Annex 3).

Tuberculin skin test to monitor changes in delayed type hypersensitivity to TB antigens will be performed at the beginning and at the end of TB treatment (week 26 visit).

As ethambutol might affect colour vision, patients’ eyes will be examined at the onset and W8 of TB treatment.

Chest X-ray will be performed at the onset of TB treatment, W8, W26 and W50 visits. In case of prolongation of TB treatment, an additional chest X-ray will be performed at the end of the treatment.

Blood tests during follow-up will aim at:

- Monitoring of the HIV infection: regular CD4+ T cell count and plasma HIV viral load. HIV viral loads will be done routinely by real time RT PCR for HIV-1 RNA plasma quantification (ANRS AC11/AC12 working group protocol coordinated by Prof. Christine

Rouzioux, Hôpital Necker Paris France) at the IPC HIV/hepatitis laboratory. The result of viral load will be available to the clinician within one month.

- Monitoring of drug side effects: transaminases at each protocol visit during TB treatment, at the week 50 visit then every 6 months.
- Monitoring of metabolic disorders: at week 50 then every 6 months, measurement of cholesterol, triglycerides and glycemia will be performed.

To measure the effectiveness of TB treatment in patients with pulmonary TB, sputum direct examination and culture (if AFB positive) will be done 2 months (week 8 visit) after the onset of TB treatment, at week 26 and at the end of the TB treatment period when TB treatment is prolonged.

Smear direct examination, culture and drug-susceptibility will also be performed in case of relapse suspicion or treatment failure.

All positive cultures on the sample taken at the onset of treatment will be evaluated for drug-susceptibility. Cultures, strain typing and drug-susceptibility will be performed at the IPC.

A genotyping of HIV strains to identify resistance mutations at the week 50 visit for the patient considered in ARV treatment failure will be performed in order to adapt the ART. In addition, genotyping on the initial sample will be done to assess primary mutation.

Study visits occuring every 6 months after week 50 will include (i) complete clinical evaluation with body weight and Karnofsky index, (ii) laboratory evaluation including CBC, AST, ALT, cholesterol/triglycerides, glycemia, CD4, viral load, HIV genotyping in case of viral load > 400 cp/mL, and (iii) AFB screening if the patient is symptomatic. In case of events, a specific patient chart will help to document the nature, the duration, the outcome and the cause of each event (e.g. death, hospitalization…). If TB recurrence is suspected, a chest X-ray and a direct examination of the sputum for AFB will be both systematically performed. Other samples will be evaluated for AFB and culture positivity based upon clinical indications (e.g. stools, adenopathy…). All AFB positive samples will be cultured at the IPC and identification/DST will be performed when the culture is positive.

7.3.2 Pharmacokinetics

The assay of efavirenz in plasma will be performed at week 4, 8, 22 and 50 among "early arm" patients and at week 10, 14, 22 and 50 among "late arm" patients.

As efavirenz is administered once daily before sleeping, blood samples will be drawn in the morning approximately 12 hours after the last dose, which will be precisely documented. After centrifugation, plasma will be separated and kept frozen until analysis.

Efavirenz will be assayed in plasma at the Faculté de Pharmacie, Université des Sciences de la Santé in Phnom Penh using a validated liquid chromatographic technique with UV detection under the supervision of Dr Anne-Marie Taburet (CHU de Bicêtre, Paris, France). The lower limit of quantification is 25 ng/ml and between run variability of quality controls is lower than 6% .

Efavirenz assay in plasma will be batch processed. Results will not be made available for patient's treatment follow-up.

7.3.3 Adverse Event

For the evaluation of safety and paradoxical reactions all adverse events occurring during the first phase of the trial will be reported on the Clinical Report Form (CRF). After W58, only WHO Stage 4 diseases (Annex 2) and events generating hospitalisation or death will be reported on CRF.

Management of drug toxicity, paradoxical reaction and new OI, according to the Cambodian National guidelines, is detailed in the MOP.

The Serious Adverse Events (SAE) will be reported to the ANRS Pharmacovigilance Department and to the DAIDS RCC Safety office according to the trial procedures described in § 6.2.2. If needed, queries on SAE will only be sent to the investigators by the ANRS Pharmacovigilance Department representative.

If needed, the treatment regimens will be adapted according to the Cambodian National guidelines and as detailed in the MOP.

7.3.4 TB treatment failure or TB relapse

Patients with culture positive strains found to be resistant to one or more first line TB agents will be changed to a customized regimen including at least three drugs to which the organism exhibits sensitivity following the guidelines of the Cambodian National Centre for TB and Leprosy Centre.

If a TB relapse is diagnosed, a second line TB treatment will be provided according to the Cambodian National guidelines and adapted for drug-susceptibility according to the MOP.

7.3.5 ARV treatment failure

Each suspicion of ART failure (see § 6.2.8) will be discussed between the investigator on site, the clinical monitor and the clinical coordinator.

As detailed in the MOP, an adjustment of the treatment will be done according to the Cambodian National Guideline.

Of note, the second line ART recommended by the Cambodian National Guideline for the Use of ARV associates two NRTI and a boosted PI.

7.3.6 Trial or treatment premature discontinuation and loss to follow-up

If a patient decides to withdraw from the trial, he/she will be requested to come for a last study examination (clinical and blood test). Various possibilities of follow-up treatment will be discussed with the patient.

If atypical mycobacterium infection is diagnosed, the patient will still be kept in the trial, followed according to the study original schedule and be part of the primary analysis performed on the study. The treatment of atypical mycobacterium infection will be provided according the Cambodian guidelines. If patients fail to appear for study site visit appointments, the clinical team on site will confidentially try to contact these patients and assist them in coming to follow-up.

7.4 Frozen samples

After completion of the study and patient's follow-up necessary assays, any quantity of blood sample left at D0, Week 8, 14, 26, 50 and after every 6 months' blood tests will be stored and frozen at -80°C at IPC. They could be used to carry out further research investigations after approval by the Scientific Advisory Board and the Cambodian Ethical Committee.

The *Mycobacterium tuberculosis* strains will also be stored at -80°C at IPC.

8 Statistical consideration

8.1 Sample size calculation

Our study assumes that 65% of co-infected patients will survive at M12 under current treatment course based on empirical observations and recent publications (9, 10). Considering that >10% increase in the survival rate in the early ARV treatment arm is clinically important, **660 patients**

will be required assuming 80% power, 5% type I error and no dropout. The calculation is based on non parametric Log-rank test for equality of survival curves using nQuery Advisor ® Software (Version 3.0, Los Angeles, CA, USA (1982).

According to the experience of the sites, loss to follow up will be less than 2%. A sensitivity analysis of the effect of a 2% loss to follow up had about a 1% effect on the power of the comparison between the two arms. With this assumption, the power of the study will reduce from 80% to 79%.

Participants will be recruited, enrolled and randomized to either the early arm or the late arm in a 1:1 allocation. Enrollment is expected to take place over a period of two years and a half (first patient enrolled in January 31st, 2006).

The accrual goal is feasible. The five selected sites are major hospitals in the country and served over 1500 HIV/TB co-infected patients in 2003. Given 30% of AFB smears will be positive we anticipate a sufficient number of eligible patients allowing 30% rooms for defaulters, loss to follow-up and refusals. Of note, the dropout rate from follow up is expected to be minimal.

8.2 Randomization procedures

The coordinating centre will randomly allocate participants in equal number to the two arms. The randomization will be stratified on study site and initial CD4 count (lower 50 cells x 10^6/l versus equal or above 50 cells x 10^6/l). The random assignment list of participants will be generated prior to the beginning of the clinical trial for each study site.

When the **investigator on site** includes one patient, he/she will phone the coordination centre team at the IPC and request the treatment arm allocation. Written confirmation will subsequently follow.

8.3 Intermediate analysis

The magnitude and the statistical significance of the treatment difference between the two arms may occur before the study ends.

As the primary analysis is based on comparison of survival rates between the two arms, several interim analyses are planned. These interim analyses will be performed according to the dates of the Data and Safety Monitoring Board (DSMB) meetings. This will allow the DSMB recommendations to be available to the Scientific Advisory Board (SAB) for discussion and rapid implementation of any decision. The "stopping rule" that will be implemented is trial stoppage if the p-value at any interim analysis is less than 0.001 (Peto and al. 1976; Slutsky and Lavery, 2004). This threshold allows us to stop the trial only when there is substantial evidence that the survival truly differs between the two arms.

8.4 Analysis plan

As an open label strategy trial, the primary analysis will be done keeping all randomized patients in the assigned treatment arm regardless of the real treatment intake (intention to treat analysis).

The analysis plan will include as follows:

- Description of the study participants according to the important features of the primary and secondary variables and of key prognostic and demographic variables.
- Description of the effect of all withdrawals from treatment and major protocol violations on the main analyses of the primary variable. Subjects lost to follow up (should any occur), withdrawn from treatment or with a severe protocol violation will be identified, and a description of them provided.
- Substantial defaults among participants will be dealt by sensitivity analyses. Results of different simulations will be discussed.
- Analysis of the results will follow the following steps:

- Comparable treatment groups by patients' characteristics
- Subgroup analyses
- Adjustment for prognostic factors
- Survival analysis using the Kaplan Meier approach and Cox's proportional hazard models to compare the two arms. Whether survival significantly differs between the two arms will be determined using the log-rank test.
- Survival analysis could also be conducted adjusting for prognostic factors
- Comparison of safety data between the two arms.

Data set will be fully analyzed based upon Intention-To-Treat principle. However, per protocol analysis restricted to the patients with confirmed tuberculosis will also be conducted and results will be compared with everyone for the full analysis.

9 Monitoring and data management

9.1 Monitoring

Monitoring will be conducted according to the Good Clinical Practice (ICH Harmonized Tripartite Guidelines for Good Clinical Practice 1996) to guarantee the good quality of the research and safeguard the health and the rights of the patient.

9.1.1 On site

The on-site trial activities (patient management; drug management and dispensation; trial monitoring) will be done under the responsibility of the **clinical coordinators**, based at the Cambodian Health Committee (CHC) in Phnom Penh. They will be responsible for coordinating all clinical activities for the trial:

- Recruitment, training and management of the clinical monitors and the trial pharmacist;
- Organising and supporting clinical patient follow-up on site;
- Supervision of trial drug management;
- Member of the steering committee;

On each investigational site, a physician known as the "**investigator**" will have the medical responsibility to conduct the trial according to the protocol.

Physicians will be recruited as **clinical monitors**. They will be responsible, under the supervision of the investigator, to assist in organizing the trial follow-up and management on site:

- Organisation of patient screening, enrollment and follow-up;
- Report clinical and biological data in the Case Report Form (CRF) according to the most updated Methodology onsite MOP that will be provided;
- Report and document all Serious Adverse Events and sent follow-up report when necessary;
- Communication and coordination with the pharmacist and coordinating centre;
- Preparation and facilitation of the monitoring visit;

The investigator and the clinical monitor must give the coordinating centre monitor access to relevant hospital or clinical records, to confirm their consistency with the CRF entries. No information about the identity of the subjects should appear on the CRF. The CRF must be completed within the week following the patient's visit. All CRF must be signed by the physician responsible of the patient follow-up.

CRF will be considered as thesource document for all the study sites regarding all the informations it contains.

A **trial pharmacist** will monitor the trial medications (ARV treatment) and other medications (TB treatment, opportunistic infection treatment) under the supervision of the clinical coordinators.

An **e**xperienced **biologist**, under the supervision of the Laboratory coordinator, is responsible for monitoring direct smear exams on each site, giving complementary training to the biologists on site and organizing quality controls.

9.1.2 Coordinating centre

The IPC Epidemiology Unit is the referent for the trial methodology and management. The IPC team is responsible, in coordination with the clinical, laboratory and administrative coordinators, for the overall trial management (preparation and organisation of the trial, monitoring, data management and analysis). The team will include the project coordinator, the coordinating methodologist, three trial monitors and one data manager.

Preparation and organisation of the trial: the IPC team will finalize and review the Case Report Forms (CRF) with all the collaborators, prepare the randomization procedures, and organize the trial reference documentation. The coordinating centre team and the clinical coordination team will prepare the operation manual describing in details the trial procedures for each collaborators (clinical and laboratory activities, treatment dispensation, data management).

Monitoring activities: the **trial monitor** will visit the site regularly during the study:

- Check the adherence to the protocol and to Good Clinical Practice (patient informed consent, protocol visit and blood test schedule…);
- Assist in the trial organisation and management on site (communication between collaborators, treatment or material availability, tracing laboratory samples follow-up and result in collaboration with the laboratory coordinator);
- Check the completeness and the accuracy of patient data on the CRF;
- Collect and check the Serious Adverse Events reporting, documentation and follow-up, and send the forms to the sponsor;
- Evaluate the progress of enrollment.

The investigator and key trial personnel must be available to assist the monitor during these visits.

DAIDS independent auditors will conduct a study audit at each site every 8 months, at IPC coordinating centre every 4 months and at the central pharmacy once a year.

Database: The trial database will be developed and maintained by the coordinating centre in collaboration with an external partner. The IPC data manager will also be in charge of the data entry and implementation of data quality insurance plans. He has to prepare daily monitoring reports and the data file necessary for the trial analysis.

Data analysis and analysis report: will be coordinated at the IPC coordinating centre.

Reporting: the operational team will be responsible for finalizing:

- the necessary reports and presentations for the Scientific Advisory Board and the DSMB meetings;
- the trial yearly progress reports;

The operational team will bring its support in preparing any communications on the trial (poster, oral presentation, papers).

9.2 Data management

9.2.1 Data collection

Data on participants will be collected on duplicated CRF during the trial. A unique CRF will be assigned for each patient. The patient will only be identified by a unique anonymous code. The

correspondence list of names/anonymous codes will be kept on site in a secured place under the responsibility of the investigator. An operation manual will be provided to help the investigators and the monitors fill out the CRF. All the information required by the protocol should be provided and any omissions require explanation.

The trial monitor from the coordinating centre will come on a regular basis to check for completeness, accuracy and legibility of data reported on the CRF. He/she will bring back the validated CRF to the coordinating centre for data entry.

9.2.2 Data entry and checking

Data will be entered at the coordinating centre (IPC). The trial database will be developed on MySQL (version 4.0.16) by a data base developer which will closely work with the coordination team.

After entry, the database will be checked for consistency. If any inconsistency or question on the data, the coordinating centre monitor will go back to the investigator for clarification during their monitoring visit. The corrections will be entered and followed in the data base.

9.3 Trial documentation storage

Essential trial documents will be retained at the coordinating centre for ten years. Data will be kept on a secured database installed on a server at the coordinating centre.

10 Trial oversight

10.1 Operational team

The operational team will monitor and coordinate the trial's daily activities on site and at the coordination centre, discuss the problems and difficulties occurring during the trial and propose solutions, write the intermediate and yearly progress report.

10.2 Scientific Advisory Board (SAB)

The SAB will meet as often as request by the coordinating investigators, after each interim analysis and at least once a year. It is in charge of:

- examination of the trial progress
- discussion of the preliminary results
- evaluation of safety data submitted through the DSMB
- propositions and final recommendations on protocol amendments
- examination of propositions of new scientific studies and of requests for use of the frozen samples before submission to the Cambodian Ethical Committee
- reviewing and giving the authorisation for any presentation, abstract, manuscript from the CAMELIA clinical trial prior submission.

10.3 Data Safety Monitoring Board (DSMB)

The DSMB will be formed specifically for the CAMELIA trial. It will be composed of several international members: physicians with an expertise in TB and in HIV infection, and methodologists.

The DSMB will examine the preliminary efficacy and safety results at each interim analysis or on any request from the SAB. The members of the committee will receive the interim analysis report by mail and conference call will be organised in order to allow discussion on the data. The mission of the committee is to check if the results justify amendment or discontinuation of the trial. Recommendations from the DSMB, which has an advisory role, will be sent to the SAB members.

11 Ethics and regulatory considerations

The study will be carried out in compliance with the protocol and in accordance with:

- the Cambodian National Ethics Committee for Health Research guidelines,
- the Declaration of Helsinki approved by the World Health Association on June 1964 amended in Tokyo 1975, Venice 1983, Hong Kong 1989, Somerset West 1996 and Edinburgh, Scotland 2000 (Annex 5),
- the recommendations of the Good Clinical Practice (ICH Harmonized Tripartite Guidelines for Good Clinical Practice E6 step 4 - 1996),
- the ANRS Ethics Charter for research in Developing Countries,
- Title 45, Part 46 of the U.S Code of Federal regulations (45 CFR 46).

11.1 Ethics Committee and Health Authorities

The protocol, the proposed information sheet to the patient and the consent form, will be submitted to the Cambodian National Ethics Committee for Health Research and to the scientific committees of ANRS and DAIDS.

The study will be implemented in Cambodia ONLY once the authorization document of the Cambodian Ministry of Health is received (Annex 6).

Once approved and authorized, the final version of the protocol will be signed by the coordinating investigators and the sponsor. All investigators will sign the protocol as an engagement to conduct the trial according to the protocol, the declaration of Helsinki, the Good Clinical Practice and adhere to the procedures described in this document.

11.2 Amendments to the protocol

Before implementation, any change or addition to this protocol requires a written protocol amendment approved by Cambodian Ethics Committee, and signed by the coordinating investigators, all the investigators and by ANRS and DAIDS.

These requirements for approval should in no way prevent any immediate action form being taken by the investigators or by the ANRS or by DAIDS in the interests of preserving the safety of all subjects included in the trial. If an immediate change to the protocol is felt to be necessary by the investigator and is implemented by him/her for safety reasons, the ANRS and DAIDS should be notified and the National Ethics Committee should be informed within 10 working days.

11.3 Disclosure and confidentiality

By signing the protocol, the investigator agrees to keep all information in strict confidence and to request similar confidentiality from his/her staff and the Cambodian National Ethics Committee for Health Research. Study documents will be stored appropriately to ensure their confidentiality.

Only physicians and scientists involved in the study, ANRS or its representatives, DAIDS or its representatives will have access to the patients' data.

All laboratory specimens, evaluation forms, reports, and other records will be identified by a coded number only to maintain subject confidentiality. All records will be kept in a locked file cabinet. All computer entry and networking programs will be done with coded numbers only.

11.4 Insurance

The ANRS will provide insurance for the whole duration of the trial for the participants (Annex 7: insurance certificate copy).

11.5 Participant benefits and risks

11.5.1 Benefits

This protocol provides opportunity for co-infected patients that are eligible for the study to have:

- the totality of their care fees paid by the study (clinical examinations, treatments, hospitalization and transportation fees);
- the proof through the smear culture that they are infected by *Mycobacterium tuberculosis*;
- access to drug susceptibility testing for *Mycobacterium tuberculosis* and to receive an adapted treatment according to these results.
- access to Atypical Mycobacterium treatment in the case of this diagnosis through the culture;
- access to viral load on a routine basis, to HIV genotyping in case of treatment failure.

Measures and insurance (i.e. agreement with NCHADS/MOH) have been taken to ensure that ARV medications are still available for all participants after the study ends. The patient's follow-up, including ARV treatment, will be done by each local clinical site as part of their activities in the “National Continuum of Care for HIV Infected Persons”.

11.5.2 Risks

Antiretroviral drugs and TB drugs may have side effects and the risk to have side effects could be enhanced by combining these two therapies as it will be the case in both arms. In addition, paradoxical reactions and IRIS may also occur in both arms at the time of concomitant HIV and TB therapy.

Participants in the early arm may experience more frequently serious side effects, more paradoxical reactions and more IRIS. The addition of 3 pills per day of ARV therapy (stavudine + lamivudine 2 pills per day + efavirenz 1 pill per day) at the beginning of the treatment might affect the adherence of the patients.

Participants in the late arm may experience the risk of worsening their HIV infection and of exposition to new OI that can be life-threatening.

12 Publications

The data analysis will be performed in the epidemiology unit at the IPC. The final analysis report which will allow preparation of publications will be send to all collaborators and to the Ethical Committee.

Any publication or oral presentation will be developed by the coordinating investigators or the operational team, and will acknowledge the ANRS and DAIDS.

The trial database is the property of ANRS and will be shared freely with DAIDS. Any data transfer should be approved by the operational team, after agreement from ANRS and DAIDS as described in the contract established between collaborators.

13 References

1. Harries AD, Hargreaves NJ, Chimzizi R, Salaniponi FM. Highly active antiretroviral therapy and tuberculosis control in Africa: synergies and potential. Bull World Health Organ 2002;80:464-9. Comment in: Bull World Health Organ 2002;80:469-70.
2. Cambodian Ministry of Health, 2002.
3. Havlir DV, Barnes PF. Tuberculosis in patients with human immunodeficiency virus infection. N Engl J Med 1999;340:367-73.
4. Dye C, Scheele S, Dolin P, Pathania V, Raviglione MC. Consensus statement. Global burden of tuberculosis: estimated incidence, prevalence, and mortality by country. WHO Global Surveillance and Monitoring Project. JAMA 1999;282:677-86.
5. Raviglione MC, Harries AD, Msiska R, WilkinsonD, Nunn P. Tuberculosis and HIV : current status in Africa. AIDS 1997; 11 (suppl B): S115-23.
6. Whalen C, Horsburgh CR Jr, Hom D, Lahart C, Simberkoff M, Ellner J. Site of disease and opportunistic infection predict survival in HIV-associated tuberculosis. AIDS 1997;11:455-60.
7. Whalen C, Horsburgh CR, Hom D, Lahart C, Simberkoff M, Ellner J. Accelerated course of human immunodeficiency virus infection after tuberculosis. Am J Respir Crit Care Med 1995;151:129-35.
8. Nunn P, Brindle R, Carpenter L, Odhiambo J, Wasunna K, Newnham R, Githui W, Gathua S, Omwega M, McAdam K. Cohort study of human immunodeficiency virus infection in patients with tuberculosis in Nairobi, Kenya. Analysis of early (6-month) mortality. Am Rev Respir Dis 1992;146:849-54.
9. S.Thai, L. Lynen, K. Kimura, J.G. Hines, S. Moung, N. Seng, W. Schrooten. Public private partnership in TB control, in Phnom Penh, Cambodia. Abstract MoPeB3188, IAS Bangkok Conference, July 2004.
10. M. Tamura, K.E. Khun, B.H. Yuos, T. Yoshiyama, K. Okada, I. Onozaki, T.E. Mao. High prevalence /incidence of TB and poor outcomes of TB treatment among people living with HIV/AIDS (PLWHA) in Phnom Penh, Cambodia. Abstract B11140, IAS Bangkok Conference, July 2004.
11. Whalen C, Okwera A, Johnson J, Vjecha M, Hom D, Wallis R, Huebner R, Mugerwa R, Ellner J. Predictors of survival in human immunodeficiency virus-infected patients with pulmonary tuberculosis. The Makerere University-Case Western Reserve University Research Collaboration. Am J Respir Crit Care Med 1996;153:1977-81.
12. Goletti D, Weissman D, Jackson RW, Graham NM, Vlahov D, Klein RS, Munsiff SS, Ortona L, Cauda R, Fauci AS. Effect of Mycobacterium tuberculosis on HIV replication. Role of immune activation. J Immunol 1996;157:1271-8.
13. Ranjbar S, Ly N, Reynes J-M, Goldfeld AE. Mycobacterium tuberculosis recall antigens inhibit HIV-1 replication in anergic donor cells via CD8+ T cell expansion and increased IL-10 levels. Submitted in 2003.
14. Stoneburner R, Laroche E, Prevots R, Singh T, Blum S, Terry P, Reatrice S, Adler J. Survival in a cohort of human immunodeficiency virus-infected tuberculosis patients in New York City. Implications for the expansion of the AIDS case definition. Arch Intern Med 1992;152:2033-7.
15. Perriens JH, Colebunders RL, Karahunga C, Willame JC, Jeugmans J, Kaboto M, Mukadi Y, Pauwels P, Ryder RW, Prignot J, et al. Increased mortality and tuberculosis treatment failure rate among human immunodeficiency virus (HIV) seropositive compared with HIV

seronegative patients with pulmonary tuberculosis treated with "standard" chemotherapy in Kinshasa, Zaire. Am Rev Respir Dis 1991;144:750-5.

16. Wallis RS, Helfand MS, Whalen CC, Johnson JL, Mugerwa RD, Vjecha M, Okwera A, Ellner JJ. Immune activation, allergic drug toxicity and mortality in HIV-positive tuberculosis. Tuber Lung Dis 1996;77:516-23.
17. Ackah AN, Coulibaly D, Digbeu H, Diallo K, Vetter KM, Coulibaly IM, Greenberg AE, De Cock KM. Response to treatment, mortality, and CD4 lymphocyte counts in HIV-infected persons with tuberculosis in Abidjan, Cote d'Ivoire. Lancet 1995;345:607-10.
18. Palella FJ Jr, Delaney KM, Moorman AC, Loveless MO, Fuhrer J, Satten GA, Aschman DJ, Holmberg SD. Declining morbidity and mortality among patients with advanced human immunodeficiency virus infection. HIV Outpatient Study Investigators. N Engl J Med 1998;338:853-60.
19. Hogg RS, Heath KV, Yip B, Craib KJ, O'Shaughnessy MV, Schechter MT, Montaner JS. Improved survival among HIV-infected individuals following initiation of antiretroviral therapy. JAMA 1998;279:450-4.
20. Burman WJ, Jones BE. Treatment of HIV-related tuberculosis in the era of effective antiretroviral therapy. Am J Respir Crit Care Med 2001;164:7-12.
21. Lopez-Cortes LF, Ruiz-Valderas R, Viciana P, *et al*. Pharmacokinetic interactions between efavirenz and rifampicin in HIV-infected patients with tuberculosis. Clin Pharmacokinet 2002; 41:681-90.
22. Narita M, Ashkin D, Hollender ES, Pitchenik AE. Paradoxical worsening of tuberculosis following antiretroviral therapy in patients with AIDS. Am J Respir Crit Care Med 1998;158:157-61.
23. Foudraine NA, Hovenkamp E, Notermans DW, Meenhorst PL, Klein MR, Lange JM, Miedema F, Reiss P. Immunopathology as a result of highly active antiretroviral therapy in HIV-1-infected patients. AIDS 1999;13:177-84.
24. Schluger NW, Perez D, Liu YM. Reconstitution of immune responses to tuberculosis in patients with HIV infection who receive antiretroviral therapy. Chest 2002;122:597-602.
25. Wendel KA, Alwood KS, Gachuhi R, Chaisson RE, Bishai WR, Sterling TR. Paradoxical worsening of tuberculosis in HIV-infected persons. Chest 2001;120:193-7.
26. Serra F, Carvalho SS, Vieira MA, Rolla V. Paradoxical reaction in patients co-infected with HIV and tuberculosis. Antiviral Therapy 2003;8(Suppl. 1):S432 [abstract].
27. Colebunders R, Lambert ML. Management of co-infection with HIV and TB. BMJ 2002;324:802-3.
28. Dean GL, Edwards SG, Ives NJ, Matthews G, Fox EF, Navaratne L, Fisher M, Taylor GP, Miller R, Taylor CB, de Ruiter A, Pozniak AL. Treatment of tuberculosis in HIV-infected persons in the era of highly active antiretroviral therapy. AIDS 2002;16:75-83.
29. Rolla V, Vieira A, Marinho F, Lourenço MC, Morgado MG. Phase IV clinical trial to access the efficacy and safety of antiretroviral treatment with ritonavir and saquinavir (400 mg - 400 mg) concomitant with rifampicin in tuberculosis and AIDS patients. Antiviral Therapy 2003;8(Suppl. 1):S432 [abstract].
30. National Cambodian guidelines for the use of antiretroviral therapy in adults and adolescents. 2007.

14 Annexes

Annex 1: Study sites

1. **Khmero-Soviet Friendship Hospital (formerly known as Preah Bath Norodom Sihanouk Hospital), Phnom Penh**

- **Pulmonary Ward:**

The ward has a capacity of 120 beds. There are 4 medical doctors, 3 medical assistants and 21 nurses from the Ministry of Health and the ward has been supported and co-managed by the CHC since 2005 in collaboration with the hospital ward chief, providing clinical, nursing and counselling support. There is an X-Ray room and a flexible bronchoscopy room in the ward. Around 100 patients are admitted per month and approximately 100 TB cases are followed up as out-patients every month. Extensive renovations of the inpatient and outpatient areas were begun in 2005 and completed in 2007 with funding from the CIPRA and the Japanese Embassy.

- **Infectious Disease Department:**

➢ **In-Patients:**

The ward has a capacity of 60 beds. There are 6 medical doctors, 2 medical assistants and 19 nurses from the Ministry of Health. Médecins sans Frontières France started a program of comprehensive care for HIV infected patients since 1997 in partnership with the Ministry of Health and NCAHDS. Around 1500 HIV infected patients are hospitalized per year in which 500 are co-infected with tuberculosis.

➢ **Out-Patients:**

In March 2007, 3500 HIV-infected patients were regularly followed, among them 2800 receiving ARVs.

2. **Calmette Hospital, Phnom Penh**

- **Ward B:**

The ward has a capacity of 55 beds. There are 6 medical doctors, 3 medical assistants and 10 nurses. CD4 count, CT scan, IRM, Xray, bronchoscopy, fibroscopy are available at Calmette hospital. The average number of HIV/AIDS cases is approximately 20 admitted per month (year 2007), and approximately 10 cases among them are having TB. Currently, there are between 5 and 10 TB cases followed-up monthly.

- **Out-Patient:**

ESTHER program (French Cooperation) in partnership with the Ministry of Health and Calmette hospital started a program of access to antiretroviral treatment in 2002. Around 1200 patients started antiretroviral treatment.

3. **CHC TB/AIDS Home care in Svay Rieng province**

- **CHC home care programme:**

The CHC TB and AIDS home care programme in Svay Rieng is based in 4 health centre zones (former district hospitals) representing a catchment's area with a population of approximately 50,000. Active case finding results in 400-500 new TB cases detected per year, of whom an estimated 5% have HIV/AIDS. In the Svay Rieng province, a total of 1711 new TB cases were notified in 2002, of whom an estimated 5% have HIV/AIDS. The CHC has successfully treated

12000 TB cases since its founding in 1994 and began AIDS prevention activities in Svay Rieng in 1997 and home based AIDS care activities in 2001.

- **Svay Rieng Referral Hospital:**

The infectious disease ward has been managed by the CHC since 2004 in collaboration with the Svay Rieng Provincial Hospital Health Team and NCHADS. The team currently consists of one medical doctor and one medical assistant together with 5 nurses. The ward is currently handling 500 HIV/AIDS out patients and among them less than 10% with TB co-infection. Voluntary and Confidential Counselling and Testing (VCCT) for HIV is organised in the hospital. X-ray and ultrasound machine are also available. The CIPRA project has included an extensive rehabilitation of the inpatient, outpatient and laboratory facilities, completed in 2006, and training of the hospital staff. Approximately 3000 patients are followed in the CHC AIDS program in Svay Rieng since its founding in 2004.

4. Chronic Diseases Clinic, Referral Hospital, Takeo

The entire hospital has a capacity of 191 beds. Voluntary and Confidential Counselling and Testing (VCCT) for HIV is organised in the hospital. Xray and ultrasound are available. The laboratory facilities are very good. The infectious disease activities are supported by Médecin Sans Frontière Belgique.

- **The chronic disease department**

There are 2 medical doctors, 3 medical assistants and 5 nurses. Around 2400 HIV infected patients are currently on follow-up and 1200 have started ARV treatment. An average of 71 newly HIV patients are diagnosed each month among them approximately 18 are co-infected with tuberculosis (13 have positive AFB sputum smear).

- **TB ward**

The ward has a capacity of 22 beds. There are 2 medical doctors. The average number of TB cases is 100 per month in average among them approximately 25 are co-infected with HIV (3 to 4 have positive AFB sputum smear).

5. Infectious Disease Ward, Siem Reap Referral Hospital

Voluntary and Confidential Counselling and Testing (VCCT) for HIV is organised in the hospital. Xray and ultrasound are available. The infectious disease activities are supported by Médecins Sans Frontières Belgium.

- **The Chronic Disease Department**

There are 4 medical doctors, 2 nurses, 2 pharmacists and 5 counsellors. Around 1800 HIV infected patients are currently on follow-up, 1300 with ARV treatment. An average of 30 newly HIV patients are diagnosed each month.

- **Infectious Diseases Ward**

The ward has a capacity of 21 beds. With around 35 admissions per month (100% HIV infected patients), the bed occupancy rate is around 95%. There are 2 medical doctors and 8 nurses from the Ministry of Health.

- **TB ward**

The ward has a capacity of 60 beds. There are 1 medical doctor and 4 nurses. The average number of TB cases is 130 per month (including outpatients); among them approximately 5 are co-infected with HIV.

Annex 2: World Health Organization staging system for HIV infection and disease

Clinical Stage 1

1. Asymptomatic infection
2. Persistent generalized lymphadenopathy
3. Acute retroviral infection

Performance Stage 1: asymptomatic, normal activity

Clinical Stage 2

4. Unintentional weight loss < 10% body weight
5. Minor mucocutaneous manifestations (e.g., dermatitis, prurigo, fungal nail infections, angular cheilitis)
6. Herpes zoster within previous 5 years
7. Recurrent upper respiratory tract infections

Performance Stage 2: symptoms, but nearly fully ambulatory

Clinical Stage 3

8. Unintentional weight loss > 10% body weight
9. Chronic diarrhea > 1 month
10. Prolonged fever > 1 month (constant or intermittent)
11. Oral candidiasis
12. Oral hairy leukoplakia
13. Pulmonary tuberculosis within the previous year
14. Severe bacterial infections
15. Vulvovaginal candidiasis

Performance Stage 3: in bed more than normal but < 50% of normal daytime during the previous month

Clinical Stage 4

16. HIV wasting syndrome
17. Pneumocystis carinii pneumonia
18. Toxoplasmosis of the brain
19. Crytosporidiosis with diarrhea > 1 month
20. Isosporiasis with diarrhea > 1 month
21. Cryptococcosis, extrapulmonary
22. Cytomegalovirus disease of an organ other than liver, spleen or lymph node
23. Herpes simplex virus infection, mucocutaneous
24. Progressive multifocal leukoencephalopathy
25. Any disseminated endemic mycois (e.g., histoplasmosis)
26. Candidiasis of the esophagus, trachea, bronchi, or lung
27. Atypical mycobacteriosis, disseminated
28. Non-typhoid Salmonella septicemia
29. Extrapulmonary tuberculosis
30. Lymphoma
31. Kaposi's sarcoma
32. HIV encephalopathy

Performance Stage 4: in bed > 50% of normal daytime during previous month

Proposed 'World Health Organization staging system for HIV infection and disease': preliminary testing by an international collaborative cross-sectional study. The WHO International Collaborating Group for the Study of the WHO Staging System. AIDS. 1993 May;7(5):711-8

Annex 3: Patients' schedule

**AntiTB treatment**	**D0** (RHZE)	**W8** (RH)	**W26**
**Early ARV treatment**	**W2**		
**Delayed ARV treatment**	**W8**		

R = randomisation (between Scr* and W2)

**Protocol Visit**	**Scr***	**R**	**W2**	**W4**	**W8**	**W10**	**W14**	**W18**	**W22**	**W26**	**W34**	**W42**	**W50**	**W58**	**W78 then every 6 mo**
Physical exam.	X		X	X	X	X	X	X	X	X	X	X	X	X	X
Tuberculin skin test	X									X					
Color vision	X				X										
Pregnancy test	X		X^{UE}		X^{UL}										
Chest X-Ray	X				X					X			X		
Blood analysis :															
CBC	X				X		X			X			X		X
ALT, AST, uricemia	X		X	X	X	X	X	X	X	X			X		X
CD4	X				X					X			X		X
Glycemia, cholesterol, triglycerides													X		X
HIV Viral load	X				X					X			X		X
Genotyping	X^1												X^1		X^1
Efavirenz assay in plasma				X^E	X^E	X^L	X^L		X				X		
Sputum direct exam					X^2					X^2					
Smear culture	X				X^3					X^3					
Mycobacterium tuberculosis drug-susceptibility	X				X^4					X^4					
Frozen plasma/serum/buffy coat	X				X		X			X			X		X

Blood samples management

**Blood Samples**	**Location**	**Scr***		**W2**	**W4**	**W8**	**W10**	**W14**	**W18**	**W22**	**W26**	**W34**	**W42**	**W50**	**W58**	**W78 then every 6 mo**
EDTA tube 5ml Hematology	**IPC**	**X**				**X**		**X**			**X**			**X**		**X**
Dry tube 5 ml Biochemistry	**IPC**	**X**		**X**	**X**	**X**	**X**	**X**	**X**	**X**	**X**			**X**		**X**
EDTA tube 5ml (HIV viral load ± genotyping[1])	**IPC**	**X^1**				**X**					**X**			**X^1**		**X^1**
EDTA 5ml tubes Pharmacokinetics	**Faculté de Pharmacie**				**X^E**	**X^E**	**X^L**	**X^L**		**X**				**X**		
**TOTAL (ml)**		**15**		**5**	**5 or 10**	**15 or 20**	**5 or 10**	**15 or 20**	**5**	**10**	**15**	**-**	**-**	**20**	**-**	**15**

* Screening

R : Randomisation; CBC : Complete Blood Count; ALT-AST : transaminases; IPC : Institut Pasteur du Cambodge.

1 : genotyping to evaluate resistance mutation will be done only for patients with detectable viral load (ART failure). For example, the genotyping will be done on the Day0 and W50 frozen plasma if viral load is detectable at week 50.

2 : only if pulmonary TB

3 : culture only if pulmonary TB with AFB positive sputum

4 : resistance of *Mycobacterium tuberculosis* will be assessed only if culture is positive

X^E : only for patients randomized in the « early arm »; X^L : only for patient randomized in the « late arm »

X^{UE} : urinary pregnancy test on site only for patients randomized in the « early arm »

X^{UL} : urinary pregnancy test on site only for patient randomized in the « late arm »

Annex 4: Information Sheet for Patients and Consent Form

What is Tuberculosis?

We have just diagnosed you and found out that you are infected with tuberculosis. Tuberculosis is an infectious disease caused by **Bacille de Koch or BK** microbe. This disease is transmitted from one person to another through coughing, sneezing and close contact activities.

You have just started receiving the treatment of tuberculosis. This treatment is very effective and can be well tolerable if the medication is correctly taken, meaning that the correct dosage of drugs is taken daily early in the morning without any food (empty stomach) for the whole 6 months. Tuberculosis treatment is divided into 2 phases: the first phase (consisting of four kinds of tuberculosis drugs for the first two months) and the second phase (consisting of two kinds of tuberculosis drugs for the last four months).

What is HIV infection and what is AIDS?

You are also infected with HIV, which can cause you to become an AIDS patient, which then you will receive treatment. AIDS is a disease caused by a virus (microbe) called HIV. This virus is spreading through out Cambodia. Approximately 3 out of 100 adults are infected with HIV. The body's defense system of HIV infected individuals gradually disappears. Generally, at first the HIV infected individuals do not notice anything, but several years later their body's defense system becomes weak and then those individuals become fragile or often get sick easily as they can no longer protect themselves from the diseases. The various diseases that the HIV infected individuals firstly catch are common illnesses (diarrhea, tuberculosis, pneumonia, etc). These illnesses can be cured if those individuals receive proper treatments at the right time. A little bit later, when the body's defense system almost disappears, the individuals will catch rare diseases which are more difficult to cure. It is the time that one is called an AIDS patient. Generally the patients will die of an incurable disease in this stage.

What are the available AIDS treatments?

Nowadays, there is still no drug to completely cure HIV infection. Once you are infected by HIV, you live with it for the rest of your life. However, there are drugs called "antiretroviral drugs" which can prevent and decrease the multiplication of HIV in the blood but cannot totally make it disappear. These antiretroviral drugs can reconstruct the body's defense system to become strong enough to protect the body against other diseases. Therefore, the risks of contracting fatal diseases related to AIDS can be greatly reduced. Then, the health condition of the patients will become better and most patients can work as usual.

These antiretroviral drugs are recommended for all HIV infected individuals whose body's defense system is too weak to protect themselves from other infections that can be contracted during AIDS stage (called 'opportunistic infections'). In order to know if you are in this stage or not, you have to undergo an analysis called "CD4 count", which quantifies the level of body's defense system in the blood. CD4 are the important cells in the body's defense system. HIV can gradually reduce the number of CD4. In Cambodia, the antiretroviral drugs are recommended when the number of CD4 is less than $250/mm^3$.

Nevertheless, these antiretroviral drugs have to be taken daily and regularly as prescribed for the whole life. Taking these drugs irregularly or incorrectly enables the virus become resistant to the drugs and leads to the failure of the treatment. The doctors can switch the drugs to ensure the

effectiveness of the treatment and to enable the patients to tolerate with this treatment. That's why the regular medical examination is necessary.

There are some drugs which can sometimes provide the undesirable or dangerous side effects. There are a lot of drugs (not only the antiretroviral drugs) which produce such side effects, but in case of antiretroviral drugs, the patients have to know these effects clearly in advance since you have to take them for the whole life. If you notice any undesirable effects, you can switch to other drugs. Hence when you accept this treatment, you will be informed about the possible side effects of the drugs and you must not hesitate to come back to your doctor whenever you feel you encounter such effects.

What are the problems that HIV infected individuals with tuberculosis face?

Generally, in Cambodia the individuals both infected with tuberculosis and HIV are in severe stage of infection, and the number of their CD4 is less than $200/mm^3$. This is a very dangerous situation for them, and the risk of contracting serious or fatal infections is even greater. In such case, the patients should start taking the antiretroviral drugs in addition to the tuberculosis treatment. Taking the antiretroviral treatment and the tuberculosis treatment together is usually effective and tolerable.

What questions might one ask?

The current medical information does not enable us to determine the exact time when it is best for an individual taking tuberculosis treatment to start antiretroviral treatment.

The late introduction of antiretroviral treatment (for example: 2 months after starting the tuberculosis treatment) is the most currently used strategy to reduce risks caused by taking the two types of drugs simultaneously. However, there is a risk that the HIV infection will be worsening and that opportunistic infections will arise, which can be fatal in some cases.

The early introduction of antiretroviral treatment (for example: 2 weeks after starting the tuberculosis treatment) may facilitate the tuberculosis treatment, reduce the risks of catching other opportunistic infections and reduce the HIV multiplication, and increase the number of CD4. However, it may increase the side effects caused by the antiretroviral drugs themselves or by the tuberculosis drugs and the interactions between the two treatments. The early introduction of antiretroviral treatment may increase the risks caused by unnoticed opportunistic infections, or the worsening of tuberculosis although the tuberculosis treatment is effective. It's obvious that with antiretroviral treatment, the body's defense system will improve. When an opportunistic infection appears, the response of this improved body's defense system is sometimes too strong, and this can worsen the individuals' medical condition. This is called Immune Restoration Syndrome. It leads to the occurrence of new symptoms and of a new opportunistic infection and can aggravate symptoms existing prior to the start of antiretroviral treatment. In relation to the tuberculosis, the following symptoms can occur:

- enlarged lymph nodes
- reoccurrence of high fever
- more severe coughing or difficult breathing

These reactions can sometimes be serious, but generally they can be treated without stopping the antiretroviral treatment and these reactions will disappear within few days to several weeks.

What is CAMELIA study?

A research study is conducted in Cambodia to determine if it is better to start antiretroviral treatment early (eg. 2 weeks after starting the tuberculosis treatment) or to wait until 2 months after starting the tuberculosis treatment. To answer such important question, we have to compare the results of the different concomitant tuberculosis treatment and the antiretroviral treatment between two groups of patients. One group has to start the antiretroviral treatment early: 2 weeks after starting the tuberculosis treatment (**Group "Early"**) and another group has to start antiretroviral treatment late: 2 months after starting the tuberculosis treatment **(Group “Late”)**. Neither you nor your doctor can decide in which group you will belong to. Instead we will use random method to decide your group. This study will be conducted under the cooperation of Cambodian, French and American medical doctors and scientists teams. This study has been approved by Cambodian Authorities and by the Cambodian Ethics Committee. 660 patients will be enrolled in the study, in which each group has 330 patients.

Each patient will be followed until the last included patient is at week 50 after initiation of TB therapy. Thus, the follow-up duration will depend on the date of your starting participating in the study. At the present time, we estimate that the end of follow-up for every patient should occur around the end of 2009.

Since you are both infected with tuberculosis and HIV, your doctor suggested you participate in this study. You have full freedom in accepting or refusing to participate in this project.

CAMELIA study participation

If you refuse to participate in this study, you can still benefit from the current treatment (including opportunistic infection prophylaxis, tuberculosis treatment and antiretroviral treatment) provided by your current doctor according to the current Cambodian National Protocol, and your medical care will not be affected by your refusal.

If you accept to participate in this study, you will sign the consent form which will also be signed by the doctor who suggested that you participate in this study. You will undergo a thorough clinical examination including color vision test, chest X-ray and tuberculin test. We will take 15 ml of your blood to do CD4 count and to do general analysis (biological testing) before giving you the antiretroviral drugs.

If your CD4 are more than 200/mm^3, you will not be included in this study. Obviously you have to continue your tuberculosis treatment for 6 months and you will still benefit from the current medical care provided by your doctor according to the current Cambodian National Protocol.

If your CD4 are less than or equal to 200/mm^3, you will be able to continue the enrollment process of this study. The randomization done by computer at the Pasteur Institute of Cambodia will assign you to either the **Group Early** or the **Group Late**. There is equal chance for you to be selected in either of the two groups. A thorough examination will be performed on your samples to

diagnose tuberculosis (culture and other tests performing to identify the efficiency of the treatment) and on your initial blood sample to do HIV viral load.

Women enrolled in CAMELIA study will have to undergo the pregnancy test before starting the antiretroviral treatment. One of the antiretroviral drugs supposed to be used at least during the first year of this study called **Efavirenz** is very dangerous for the fetus and is not recommended for pregnant women. You must be aware that you are not allowed to participate in this study if you are pregnant or breastfeeding. Moreover, sexually active women have to use two effective contraceptive methods (at least one of the methods is the use of condom appropriately) until they finish Efavirenz therapy (and at least six weeks after the end of the **Efavirenz** therapy). Your doctor will give you all advice regarding the use of contraceptive methods available for you. All the methods you choose are free of charge. If you are pregnant while taking **Efavirenz**, you should contact your doctor as soon as possible to discuss with him/her and to modify the treatment if necessary (for example: suspending **Efavirenz**)

Before introducing antiretroviral treatment, a lot of informational, awareness assessment and counseling sessions will be carried out so that you can be well prepared for this treatment. Your understanding of the HIV infection, of all issues related to antiretroviral treatment, and your personal commitment is absolutely crucial for the long-term success of this treatment.

The initial antiretroviral treatment is composed of three antiretroviral drugs including **D4T** or **Stavudine**, **3TC** or **Lamivudine** and **Efavirenz.** During the first year of the study, ARVs are provided by the study sponsor. After this first year, they will be provided per the Cambodian National Program (NCHADS). Every ARV used are prequalified per national and international procedures (e.g. WHO). At any moment during the study period, your ARV treatment will be adapted by your doctor according to your health status and/or blood test results.

In addition to the tuberculosis treatment (taking the drugs daily in the morning for 6 months) and the antiretroviral treatment, you also have to take the following drugs:

- Cotrimoxazole 480mg 2 tablets/day for prophylaxis of Pneumocytosis, which is a kind of severe lung disease, when your CD4 are less than $200/mm^3$.
- Fluconazole 1 tablet/day for prophylaxis of Cryptococcosis meningitis, which is a kind of frequent meningitis in Cambodia, when your CD4 is less than $100/mm^3$.

During the first year of the study, you will attend at least 12 visits and get your blood checked at least 10 times. Such amount of the blood specimens is not dangerous for the patients at all. It just sometimes can cause some discomforts such as bleeding or bruising, dizziness, or needle related-lesion. Afterwards, you will receive an every 6 months' follow-up including medical check-up and blood test.

During the whole study period, you can come to the hospital whenever you have any health problems (intolerance of the treatment, occurrences of other infections, more severe tuberculosis or injury related to the study etc.). In such cases, your doctor will suggest you undergo additional tests if needed (for example biological analysis, chest X-ray…). The study will pay for all costs (additional tests, hospitalization or doctor's fee, treatments, travel fees…). In case of injury related to the study happens, the insurance taken by ANRS will cover related medical care.

During the study period, the adherence to tuberculosis and the antiretroviral treatments will be evaluated during the dispensations of drugs, meaning that you will be requested to return the remaining dispensed drugs.

Detailled CAMELIA follow-up:

Two weeks after starting the tuberculosis treatment (**week 2**), a clinical examination and biological check-up (by taking 5ml of your blood specimen) will be done to determine whether the patients can tolerate with the tuberculosis treatment or not. Antiretroviral treatment drugs will be provided to the patients randomized in the Group Early. The female patients in **Group Early** will have to undergo the urinary pregnancy test before they start taking the antiretroviral drugs.

Two weeks later (**week 4**), another clinical examination and the biological check-up (by taking 5 ml of your blood specimen) will be done again to confirm the tolerance of the tuberculosis treatment. For the patients who started the antiretroviral treatment (**Group Early**) will be taken 5 ml of their blood specimen to measure the quantity of Efavirenz remaining in the blood.

One month later (**week 8**), equal to 2 months after the beginning of the tuberculosis treatment, the patients will undergo a clinical examination (including color vision test), chest X-ray and complete biological tests (15 ml of blood specimen needed), and Efavirenz blood measurement (5 ml of blood specimen needed) for the patients in **Group Early**. Direct sputum examination will be performed for pulmonary tuberculosis patients. Moreover, the tuberculosis treatment will be changed (the beginning of the second phase of the treatment). The patients who have not yet begun the antiretroviral treatment (**Group Late**) will start taking the antiretroviral drugs this day. The female patients in **Group Late** will have to undergo the urinary pregnancy test before they start taking the antiretroviral drugs.

Two weeks later (**week 10**), the patients will undergo another clinical examination and biological analysis (5 ml of blood specimen needed) to assess the extent the patients can tolerate with both the tuberculosis and antiretroviral treatments. The patients in Group Late will be taken 5 ml of the blood specimen to measure the quantity of Efavirenz remaining in their blood.

After that, the following tests will be done monthly by the means of clinical examination and blood analysis (5 to 10 ml of blood specimen needed) until the end of the tuberculosis treatment. The patients in Group Late will be taken another 5ml of the blood specimen after they have taken the antiretroviral drugs for 6 weeks (**week 14**) to measure the quantity of Efavirenz remaining in their blood. However, all the patients in both groups will be taken 5 ml of the blood specimen in the following 8 weeks to measure the quantity of Efavirenz remaining in their blood (**week 22**).

The tuberculosis treatment usualy finishes in the sixth month (**week 26**). At that time the researchers will do a clinical check-up, a chest X-ray, a complete biological testing (15 ml of blood specimen needed) and tuberculin test. In addition, the direct sputum examination will be done for the pulmonary Tuberculosis patients.

In most of the cases, you will be considered as cured from your TB at the end of your TB treatment. In the very rare case of tuberculosis treatment failure, a new tuberculosis treatment will be given to you based on the results of the drugs sensitivity test.

Several months after the end of your TB treatment, it might rarely happen that you are suspected of getting a new TB. In this case, a clinical check-up, a blood collection (15 ml), a chest X-ray and some samples collections dedicated to find the TB agent will be performed. If a new TB is effectively diagnosed, a new treatment will be started.

From **week 26** until **week 58**, the patients will be checked every two months (**week 34, 42, 50 and 58**). At **week 50**, a clinical check-up, chest X-ray, a complete biological analysis and Efavirenz blood measurement (20ml of blood specimen needed) will be performed.

After W58, next visit will be W78 (one year and half after your enrollement in the study). At W78 and at every following protocol visit which will occur every 6 months, a clinical check-up and a complete biological analysis (15ml of blood) will be performed.

All antiretroviral drugs can produce side effects. Some side effects may be mild and will disappear when you have taken the antiretroviral drugs for a few weeks, or they may become severe (yet rarely life-threatening) which requires treatment or hospitalization. It is very important for you to be aware of it, and you will have to come back to your doctor whenever you are sick so that the doctor can identify the cause of your illness and cure it.

The table below shows the examples of frequent side effects caused by the antiretroviral drugs. However this list does not mention all possible side effects:

Examples of Mild side effects	Examples of Severe side effects
1. Loss of appetite<br>2. Stomachache<br>3. Vomiting and nausea<br>4. Headache<br>5. Dizziness<br>6. Changes in mood or sleep (bad dreams)<br>7. Tiredness & Feeling of weakness<br>8. Abdominal pain and diarrhea<br>9. Numbness, tingling or pain in your hands or feet<br>10. Body fat changes	1. Skin rash<br>2. Liver problems<br>3. Pancreas problems<br>4. Blood acidosis<br>5. Fetal abnormalities<br>6. Anemia<br>7. Severe mental diseases (depression, psychosis)

In rare cases, some patients taking antiretroviral drugs can develop a condition called "*lactic acidosis*" whose symptoms include unexplained weight loss, stomach problems, anorexia, nausea, vomiting, fatigue, muscle weakness (paralysis), cramps, muscle pain, shortness of breath, and severe liver problems, which can be fatal in some cases. Severe liver problems and death occur more often in female patients and patients with concomitant liver disease.

Antiretroviral drugs can cause changes in body fat distribution, which can modify the shape of the patients' body such as increase the size of the stomach area, the waist, the area behind the neck and the breasts; and thinness of the face, arms and legs. The use of antiretroviral drug combinations may also be associated with changes in lipids in the blood.

Taking alcohol, or mind-or mood-altering drugs with drugs of the study can make the side effects worse.

When the doctor of the study gives you the study drugs, he/she will tell you the likely side effects of the drugs. During the study period, you will be told all these side effects, particularly if you receive new antiretroviral drugs. If you have any questions related to the side effects of the drugs prescribed during this study, you can ask the study staff.

If you stop using one or more drugs, your health could be worsening, especially if you are infected by an unknown virus that can cause liver disease.

In case of antiretroviral treatment failure, a test to find virus resistances (viral genotyping) will be performed on the blood specimens, kept at the Pasteur Institute of Cambodia. Then, a new antiretroviral treatment will be proposed to you based on the results of this test.

During the study period we will inform you about all update information related to this study or other studies which may affect your health, welfare or your willingness to stay in the study. You can withdraw from this study whenever you want without any consequences on your medical follow-up. A last visit will then be done. Moreover, your participation in the study can be stopped by your doctor if he thinks it is necessary. National Health Authorities or the Scientific Committee who monitors this study may also decide to end it. In any cases mentioned above, your treatment will be continued or adapted to your own needs and the treatment will be provided as initially planned by the study.

All your personal information collected during the study period (data related to clinical check-up, biological testing, X-ray) will be kept with the data collected from other participating patients. These data will be registered anonymously in a computerized database. The doctors and scientists conducting the study, sponsors and their representatives, and related national medical authority(ies) can have access to your medical record on the condition that they respect its confidentiality. If the result of the CAMELIA study is published, your identity will be kept confidentially.

Your blood specimens will be frozen and kept anonymously in the fridge of the Pasteur Institute of Cambodia located in Phnom Penh. These blood specimens may be used for additional tests

including genomic analysis as required by any future studies agreed upon by the Cambodian Ethics Committee.

During the study period, you can ask any questions or request for additional information from your doctor or the responsible person of the study at the hospital you go to. It is Dr………………………… (Tel: ……………….).

What happens when the study ends?

After this study ends, your medical care will be ensured continuatively at the same hospital with the same condition to the other HIV/AIDS patients as a part of Cambodian National Program. Thus, your treatment will still be provided after the end of the study.

What are the advantages and disadvantages of participating in CAMELIA study?

At the moment we do not know yet if it is better to begin the antiretroviral treatment early rather than late. Therefore, we cannot tell you whether the treatment provided to you (taking the antiretroviral drugs 2 weeks or 2 months after starting the anti-tuberculosis drugs) is better. In addition, we cannot tell you as well in which case the concomitant tuberculosis and antiretroviral treatment is more effective and in which case the side effects are less. Only the final results of this study that can give us the answer.

However, we can actually tell you that when you participate in this study:

- You will contribute to the answer of when the best time is for the HIV infected patients whose body's defense system is very weak (CD4 are less than 200/mm^3) to start taking antiretroviral treatment while they are taking the anti-tuberculosis drugs. It is a very crucial answer for all the HIV/AIDS patients in Cambodia as well as in Asia and the whole world.
- You will benefit from the thorough examinations related to Tuberculosis particularly:
    - There will be clear evidences to prove that you are really infected with Tuberculosis and not with others diseases (Mycobacterium that is not BK) which are difficult to distinguish from Tuberculosis. If you are found out to be infected with Mycobacterium (which is not BK) the treatment will be changed.
    - Sensitivity test of tuberculosis germs to various anti-tuberculosis drugs used in the treatment, which is not a routine test, will be performed. In case of tuberculosis resistance, you will be provided the appropriate treatment.

You will benefit from thorough examinations to monitor your antiretroviral treatment, especially the viral load in the blood. In case of antiretroviral failure, research on the viral resistance can help us modify your treatment. These tests are not performed in routine.

CAMELIA study Informed Consent

I, (full name) ……………………, the undersigned declare that I have read or have been read the Information Notice on CAMELIA Study attached with this form and clearly understood the objectives, the advantages and disadvantages of the CAMELIA study. I have the opportunity to ask all questions that I want to Dr. (full name)……………………….. who suggested my participation in this study as described in the Information Notice on CAMELIA study.

I have also fully understood that I have full freedom to accept or refuse to participate in this CAMELIA study. Once I am included in this study, I can also withdraw from it whenever I want without any problems and I will continue to benefit from the regular medical care and on-going check-up.

Therefore, I personally agree to participate in this research study under the conditions mentioned in this document, with full knowledge without any pressure from any medical staff.

I allow my confidential data be used and analyzed by all personnel involved in this study who must respect its medical confidentiality.

Done at………….. Date:

Signature

I, Dr. …………………………….., the undersigned declare that I have explained the patient whose name is written above about the issues related to the study, types of inclusion into the study, and the monitoring on the CAMELIA study. I solemnly promise that I will respect all the terms and conditions mentioned in this consent form by keeping it confidentially and respecting the individual's rights and freedom as well as the requirements of the scientific work.

Done at………….. Date:

Signature:

Annex 5: Helsinki declaration

WORLD MEDICAL ASSOCIATION DECLARATION OF HELSINKI

Ethical Principles for

Medical Research Involving Human Subjects

Adopted by the 18th WMA General Assembly
Helsinki, Finland, June 1964
and amended by the
29th WMA General Assembly, Tokyo, Japan, October 1975
35th WMA General Assembly, Venice, Italy, October 1983
41st WMA General Assembly, Hong Kong, September 1989
48th WMA General Assembly, Somerset West, Republic of South Africa, October 1996
and the
52nd WMA General Assembly, Edinburgh, Scotland, October 2000

A. INTRODUCTION

1. The World Medical Association has developed the Declaration of Helsinki as a statement of ethical principles to provide guidance to physicians and other participants in medical research involving human subjects**.** Medical research involving human subjects includes research on identifiable human material or identifiable data.
2. It is the duty of the physician to promote and safeguard the health of the people. The physician's knowledge and conscience are dedicated to the fulfilment of this duty.
3. The Declaration of Geneva of the World Medical Association binds the physician with the words, "The health of my patient will be my first consideration," and the International Code of Medical Ethics declares that, "A physician shall act only in the patient's interest when providing medical care which might have the effect of weakening the physical and mental condition of the patient."
4. Medical progress is based on research which ultimately must rest in part on experimentation involving human subjects.
5. In medical research on human subjects, considerations related to the well-being of the human subject should take precedence over the interests of science and society.
6. The primary purpose of medical research involving human subjects is to improve prophylactic, diagnostic and therapeutic procedures and the understanding of the aetiology and pathogenesis of disease. Even the best proven prophylactic, diagnostic, and therapeutic methods must continuously be challenged through research for their effectiveness, efficiency, accessibility and quality.
7. In current medical practice and in medical research, most prophylactic, diagnostic and therapeutic procedures involve risks and burdens.
8. Medical research is subject to ethical standards that promote respect for all human beings and protect their health and rights. Some research populations are vulnerable and need special protection. The particular needs of the economically and medically disadvantaged must be recognized. Special attention is also required for those who cannot give or refuse consent for themselves, for those who may be subject to giving consent under duress, for those who will not benefit personally from the research and for those for whom the research is combined with care.
9. Research Investigators should be aware of the ethical, legal and regulatory requirements for research on human subjects in their own countries as well as applicable international requirements. No national ethical, legal or regulatory requirement should be allowed to reduce or eliminate any of the protections for human subjects set forth in this Declaration.

B. BASIC PRINCIPLES FOR ALL MEDICAL RESEARCH

10. It is the duty of the physician in medical research to protect the life, health, privacy, and dignity of the human subject.

11. Medical research involving human subjects must conform to generally accepted scientific principles, be based on a thorough knowledge of the scientific literature, other relevant sources of information, and on adequate laboratory and, where appropriate, animal experimentation.
12. Appropriate caution must be exercised in the conduct of research which may affect the environment, and the welfare of animals used for research must be respected.
13. The design and performance of each experimental procedure involving human subjects should be clearly formulated in an experimental protocol. This protocol should be submitted for consideration, comment, guidance, and where appropriate, approval to a specially appointed ethical review committee, which must be independent of the investigator, the sponsor or any other kind of undue influence. This independent committee should be in conformity with the laws and regulations of the country in which the research experiment is performed. The committee has the right to monitor ongoing trials. The researcher has the obligation to provide monitoring information to the committee, especially any serious adverse events. The researcher should also submit to the committee, for review, information regarding funding, sponsors, institutional affiliations, other potential conflicts of interest and incentives for subjects.
14. The research protocol should always contain a statement of the ethical considerations involved and should indicate that there is compliance with the principles enunciated in this Declaration.
15. Medical research involving human subjects should be conducted only by scientifically qualified persons and under the supervision of a clinically competent medical person. The responsibility for the human subject must always rest with a medically qualified person and never rest on the subject of the research, even though the subject has given consent.
16. Every medical research project involving human subjects should be preceded by careful assessment of predictable risks and burdens in comparison with foreseeable benefits to the subject or to others. This does not preclude the participation of healthy volunteers in medical research. The design of all studies should be publicly available.
17. Physicians should abstain from engaging in research projects involving human subjects unless they are confident that the risks involved have been adequately assessed and can be satisfactorily managed. Physicians should cease any investigation if the risks are found to outweigh the potential benefits or if there is conclusive proof of positive and beneficial results.
18. Medical research involving human subjects should only be conducted if the importance of the objective outweighs the inherent risks and burdens to the subject. This is especially important when the human subjects are healthy volunteers.
19. Medical research is only justified if there is a reasonable likelihood that the populations in which the research is carried out stand to benefit from the results of the research.
20. The subjects must be volunteers and informed participants in the research project.
21. The right of research subjects to safeguard their integrity must always be respected. Every precaution should be taken to respect the privacy of the subject, the confidentiality of the patient’s information and to minimize the impact of the study on the subject's physical and mental integrity and on the personality of the subject.
22. In any research on human beings, each potential subject must be adequately informed of the aims, methods, sources of funding, any possible conflicts of interest, institutional affiliations of the researcher, the anticipated benefits and potential risks of the study and the discomfort it may entail. The subject should be informed of the right to abstain from participation in the study or to withdraw consent to participate at any time without reprisal. After ensuring that the subject has understood the information, the physician should then obtain the subject's freely given informed consent, preferably in writing. If the consent cannot be obtained in writing, the non-written consent must be formally documented and witnessed.
23. When obtaining informed consent for the research project the physician should be particularly cautious if the subject is in a dependent relationship with the physician or may consent under duress. In that case the informed consent should be obtained by a well-informed physician who is not engaged in the investigation and who is completely independent of this relationship.

24. For a research subject who is legally incompetent, physically or mentally incapable of giving consent or is a legally incompetent minor, the investigator must obtain informed consent from the legally authorized representative in accordance with applicable law. These groups should not be included in research unless the research is necessary to promote the health of the population represented and this research cannot instead be performed on legally competent persons.
25. When a subject deemed legally incompetent, such as a minor child, is able to give assent to decisions about participation in research, the investigator must obtain that assent in addition to the consent of the legally authorized representative.
26. Research on individuals from whom it is not possible to obtain consent, including proxy or advance consent, should be done only if the physical/mental condition that prevents obtaining informed consent is a necessary characteristic of the research population. The specific reasons for involving research subjects with a condition that renders them unable to give informed consent should be stated in the experimental protocol for consideration and approval of the review committee. The protocol should state that consent to remain in the research should be obtained as soon as possible from the individual or a legally authorized surrogate.
27. Both authors and publishers have ethical obligations. In publication of the results of research, the investigators are obliged to preserve the accuracy of the results. Negative as well as positive results should be published or otherwise publicly available. Sources of funding, institutional affiliations and any possible conflicts of interest should be declared in the publication. Reports of experimentation not in accordance with the principles laid down in this Declaration should not be accepted for publication.

C. ADDITIONAL PRINCIPLES FOR MEDICAL RESEARCH COMBINED WITH MEDICAL CARE

28. The physician may combine medical research with medical care, only to the extent that the research is justified by its potential prophylactic, diagnostic or therapeutic value. When medical research is combined with medical care, additional standards apply to protect the patients who are research subjects.
29. The benefits, risks, burdens and effectiveness of a new method should be tested against those of the best current prophylactic, diagnostic, and therapeutic methods. This does not exclude the use of placebo, or no treatment, in studies where no proven prophylactic, diagnostic on therapeutic method exists.
30. At the conclusion of the study, every patient entered into the study should be assured of access to the best proven prophylactic, diagnostic and therapeutic methods identified by the study.
31. The physician should fully inform the patient which aspects of the care are related to the research. The refusal of a patient to participate in a study must never interfere with the patient physician relationship.
32. In the treatment of a patient, where proven prophylactic, diagnostic and therapeutic methods do not exist or have been ineffective, the physician, with informed consent from the patient, must be free to use unproven or new prophylactic, diagnostic and therapeutic measures, if in the physician's judgement it offers hope of saving life, re-establishing health or alleviating suffering. Where possible, these measures should be made the object of research, designed to evaluate their safety and efficacy. In all cases, new information should be recorded and, where appropriate, published. The other relevant guidelines of this Declaration should be followed.

Annex 6: Ethic committee approval and Ministry of Health Authorization

1. **Version 3.0 October 22, 2004 approved by the Cambodian "National Ethic Committee for Health Research" on December 3rd, 2004.**

KINGDOM OF CAMBODIA
NATION - RELEGION - KING

MINISTRY OF HEALTH
National Ethics Committee for Health Research
No086.NECHR...

Phnom Penh, December 6th , 2004

Dr. Sok Thim
Principal Investigator,
Project: Early vs late introduction of antiretroviral therapy in naive HIV-infected adult patients with tuberculosis in Cambodia

Subject: Ethical approval

Reference: December 3rd, 2004 NEC meeting minute

Dear Dr. Thim,

I am please to notify you that your project entitled "Early vs late introduction of antiretroviral therapy in naive HIV-infected adult patients with tuberculosis in Cambodia" has been approved by the National Ethics Committee for Health Research, Cambodia on December 3rd, 2004.

The principal investigator of the project shall submit a copy of the progress and final report to the committee's secretariat at the National Institute of Public Health at #2 Kim Il Sung Blvd., Khan Tuol Kok, Phnom Penh, Cambodia (Tel: 855-23-880-345, Fax: 855-23-880-346 and email: research03@online.com.kh).

Regards,

For Chairman

H.E. Prof. ENG HUOT

2. **Version 4.0 August 5, 2005 approved by the Cambodian "National Ethic Committee for Health Research" on August 19th, 2005.**

ព្រះរាជាណាចក្រកម្ពុជា
KINGDOM OF CAMBODIA
ជាតិ សាសនា ព្រះមហាក្សត្រ
NATION RELIGION KING

ក្រសួងសុខាភិបាល
MINISTRY OF HEALTH
គណៈកម្មាធិការជាតិក្រមសីលធម៌
របស់ការស្រាវជ្រាវសុខភាពដែលទាក់ទងនឹងមនុស្ស
National Ethics Committee for Health Research

លេខ ០៤៦ NECHR........

រាជធានីភ្នំពេញ, ថ្ងៃទី ០៥ ខែ ៩ ឆ្នាំ200៥...

Dr. Sok Thim
Principal Investigator,

Project: Early vs late introduction of antiretroviral therapy in naive HIV-infected adult patients with tuberculosis in Cambodia.

Subject: Ethical approval

Reference: August 19th, 2005 NEC meeting minute

Dr. Sok Thim

I am please to notify you that your project entitled " Early vs late introduction of antiretroviral therapy in naive HIV-infected adult patients with tuberculosis in Cambodia." has been approved by the National Ethics Committee for Health Research, Cambodia on August 19th, 2005.

The principal investigator of the project shall also submit a copy of the progress and final report to the committee's secretariat at the National Institute of Public Health at #2 Kim Il Sung Blvd., Khan Tuol Kok, Phnom Penh, Cambodia (Tel: 855-23-880-345, Fax: 855-23-880-346 and email: research03@online.com.kh).

Regards,

Chairman

H.E. Prof. ENG HUOT

លេខ ២ មហាវិថី គីម អ៊ិលស៊ុង . ខ័ណ្ឌទួលគោក . រាជធានីភ្នំពេញ . ទូរស័ព្ទ (៨៥៥-២៣) ៨៨០ ៣៤៥ . ទូរស័ព្ទដៃ (៨៥៥-១២) ២៨០ ៧៩០ . (៨៥៥-១២) ៨៤២ ៤៤២
2 Blvd KIM YL SUNG, Khan Toul Kork, Phnom Penh , Tel : (855-23) 880 345 , Mobile phone : (855-12) 280 790, (855-12) 842 442

3. **Version 5.0 December 22nd, 2005 approved by the Cambodian "National Ethic Committee for Health Research" on December 30th, 2005.**

ព្រះរាជាណាចក្រកម្ពុជា
KINGDOM OF CAMBODIA
ជាតិ សាសនា ព្រះមហាក្សត្រ
NATION RELIGION KING

ក្រសួងសុខាភិបាល
MINISTRY OF HEALTH
គណៈកម្មាធិការជាតិក្រមសីលធម៌
របស់ការស្រាវជ្រាវសុខភាពដែលទាក់ទងនឹងមនុស្ស
National Ethics Committee for Health Research

លេខ 043 NEC

រាជធានីភ្នំពេញ, ថ្ងៃទី 05 ខែ 01 ឆ្នាំ200 6

Dr. Sok Thim
Principal Investigator,

Project: Early vs. late introduction of antiretroviral therapy in naive HIV-infection adult patients with tuberculosis in Cambodia

Subject: Ethical approval
Reference: December 30th,2005 NEC meeting minute

Dear Dr. Sok Thim

I am writing to notify you that your project entitled "Early vs. late introduction of antiretroviral therapy in naive HIV-infection adult patients with tuberculosis in Cambodia " has been approved by the National Ethics Committee for Health Research in the meeting on December 30th,2005. However, we required that the ARV drug that will be used in the study should be on the most updated WHO pre-qualified ARV list.

The principal investigator of the project shall submit a copy of the progress and final report to the committee's secretariat the National Institute of Public Health #2 Kim Il Sung Blvd, Khan Tuol Kok, Phnom Penh, Cambodia (Tel: 855-23-880-345, Fax: 855-23-880-346)

Regards,

Chairman

H.E. Prof. ENG HUOT

លេខ ២ មហាវិថី គីម អ៊ិលស៊ុង . ខណ្ឌទួលគោក . រាជធានីភ្នំពេញ . ទូរស័ព្ទ (៨៥៥-២៣) ៨៨០ ៣៤៥ . ទូរស័ព្ទដៃ (៨៥៥-១២) ២៨០ ៧៩០ . (៨៥៥-១២) ៨៤២ ៤៤២
2 Blvd KIM YL SUNG, Khan Toul Kork, Phnom Penh , Tel : (855-23) 880 345 , Mobile phone : (855-12) 280 790, (855-12) 842 442

Annex 7: Insurance

Biomedicinsure
Société de courtage d'Assurances
SAS au capital de 48.000 €
RCS VANNES B 347 531 089 - APE 672Z
PARC D'INNOVATION BRETAGNE SUD
C.P. 142 - 56038 VANNES CEDEX
Tel 33 2 97 69 19 19 - Fax 33 2 97 69 11 11
E-mail : biomail@biomedic-insure.com

GERLING FRANCE

INSURANCE CERTIFICATE

90791200500009

We the undersigned GERLING -Allgemeine Versicherungs AG - Direction for the France - 111 rue de Longchamp 75116 PARIS, certify thereby that the company :

STE ANRS
101 RUE DE TOLBIAC
75013 PARIS

is insured by policy n° **16.8090791** for the liability as sponsor or any participating parties for bodily injuries caused to persons subjects to clinical investigations and related to the investigations with an indemnity limited to **160.000 €** per victim, **800.000 €** per protocol and **1.600.000 €** per year of insurance.

The policy n° **16.8090791** applies to the following clinical trial :

Introduction précoce versus tardive d'un traitement antirétroviral chez des patients adultes atteints de tuberculose et co-infectés par le VIH au Cambodge.
CAMELIA
Protocol ANRS 1295

THE PRESENT CERTIFICATE DOES NOT ENGAGE THE INSURER BEYOND THE LIMITS OF POLICY IT IS REFERING TO.

Paris, le 14 octobre 2005

BIOMEDIC INSURE.
PARC D'INNOVATION BRETAGNE SUD
CP 142
56038 VANNES CEDEX

Biomedicinsure
Société de courtage d'Assurances
Roland MARCA
RCS VANNES [illegible] APE 672Z
PARC D'INNOVATION BRETAGNE SUD
C.P. 142 - 56038 VANNES CEDEX
Tel 33 2 97 69 19 19 - Fax 33 2 97 69 11 11
E-mail : biomail@biomedic-insure.com

GERLING
Allgemeine Versicherungs-AG
Direction pour la France
Marie-France Hurel

24/41

Direction pour la France
111, rue de Longchamp
75116 PARIS
Téléphone : +33 (0) 1 44 05 56 00
Téléfax : +33 (0) 1 44 05 56 66
e-mail : info@gerling.fr
Web : www.gerling.fr

Entreprise privée régie par le Code des Assurances
Capital 224 789 463 €
R.C.S. Paris B 775 746 480

Siège social : Gerling-Konzern Allgemeine Versicherungs-AG,
Von-Werth-Strasse, 4-14, D - 50670 Köln
Téléphone : 00 49 221 144 1
Telefax : 00 49 221 144- 33 19
